# Sub-Nyquist artefacts and sampling moiré effects

**DOI:** 10.1098/rsos.140550

**Published:** 2015-03-18

**Authors:** Isaac Amidror

**Affiliations:** Ecole Polytechnique Fédérale de Lausanne (EPFL), Lausanne 1015, Switzerland

**Keywords:** sampling, reconstruction, moiré effects, sub-Nyquist artefacts, sampling theorem

## Abstract

Sampling moiré effects are well known in signal processing. They occur when a continuous periodic signal *g*(*x*) is sampled using a sampling frequency *f*_s_ that does not respect the Nyquist condition, and the signal-frequency *f* folds over and gives a new, false low frequency in the sampled signal. However, some visible beating artefacts may also occur in the sampled signal when *g*(*x*) is sampled using a sampling frequency *f*_s_ which fully respects the Nyquist condition. We call these phenomena *sub-Nyquist artefacts*. Although these beating effects have already been reported in the literature, their detailed mathematical behaviour is not widely known. In this paper, we study the behaviour of these phenomena and compare it with analogous results from the moiré theory. We show that both sampling moirés and sub-Nyquist artefacts obey the same basic mathematical rules, in spite of the differences between them. This leads us to a unified approach that explains all of these phenomena and puts them under the same roof. In particular, it turns out that all of these phenomena occur when the signal-frequency *f* and the sampling frequency *f*_s_ satisfy *f*≈(*m*/*n*)*f*_s_ with integer *m*, *n*, where *m*/*n* is a reduced integer ratio; cases with *n*=1 correspond to true sampling moiré effects.

## 2. Introduction

**Figure 1.**
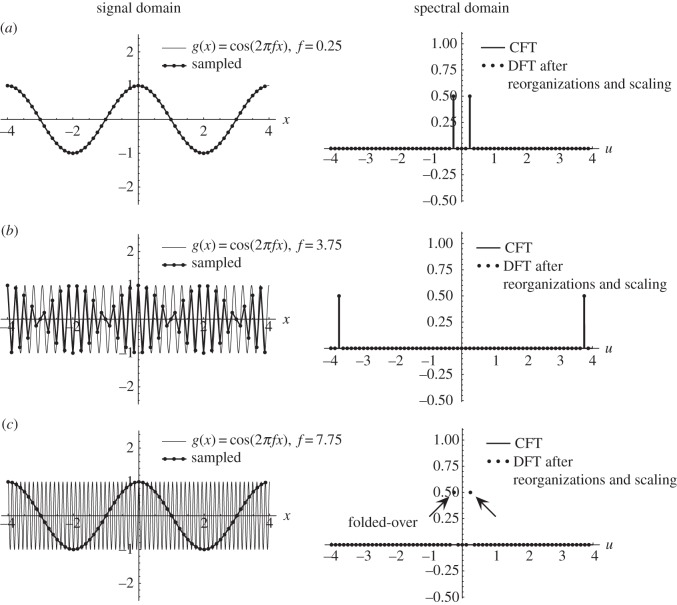
Comparison between (*a*) a case with no sampling artefacts, (*b*) a case with a sub-Nyquist artefact, and (*c*) a case with a true sampling moiré effect. Each row shows in the left-hand panel a continuous signal *g*(*x*)=*cos*(2*πfx*) having a different frequency *f*, as well as its sampled version after being sampled with a sampling frequency of *f*_s_=8 (i.e. with a sampling interval of Δ*x*=1/*f*_s_=1/8). For the sake of clarity, consecutive samples (dots) are connected by straight line segments; the original, continuous cosines are drawn by thinner curves. The right-hand panels show the respective continuous Fourier transform (CFT) of the continuous signal *g*(*x*), along with the discrete Fourier transform (DFT) of its sampled version, after having applied the required reorganizations and scalings. The only difference between the rows is in the frequency *f* of the original signal *g*(*x*): (*a*) *f*=0.25, (*b*) *f*=3.75, and (*c*) *f*=7.75. In (*a*), no aliasing occurs, and the sampled signal looks similar to the original signal. In (*c*), the cosine frequency *f* already exceeds (1/2)*f*_s_, meaning that aliasing does exist (note that the corresponding impulses in the DFT are folded over: they exceed the boundaries of the DFT spectrum, −(1/2)*f*_s_…(1/2)*f*_s_, and re-enter from the opposite end). When such folded-over (aliased) impulses fall close to the spectrum origin, as in row (*c*), a strong low-frequency sampling moiré becomes more prominent than the original cosine function itself. On the other hand, the low-frequency beating effect which appears in the signal domain in row (*b*) is not a true moiré: it occurs when the frequency *f* of the cosine signal being sampled is below the Nyquist limit (1/2)*f*_s_, so that no aliasing may occur; and unlike in row (*c*), no corresponding low-frequency impulses appear in its spectrum. This beating effect is a sub-Nyquist artefact. A more detailed explanation of cases (*c*) and (*b*) and the differences between them is provided in figures 2 and 3, respectively, and in §3. It turns out that all these phenomena occur when *f*≈(*m*/*n*)*f*_s_ with integer *m*, *n*; cases with *n*=1 correspond to true sampling moiré effects.

According to the classical sampling theorem, all the information in a continuous signal *g*(*x*) is preserved in its sampled version *g*(*x*_*k*_) if the sampling frequency *f*_s_ respects the Nyquist condition, i.e. if *f*_s_ is at least twice the highest frequency contained in *g*(*x*).^1^ When sampling a continuous periodic signal *g*(*x*) using a sampling frequency *f*_s_ that does not respect the Nyquist condition, various moiré or aliasing artefacts may appear in the resulting sampled signal *g*(*x*_*k*_) (e.g. figure 1*c*). Nevertheless, it is also known (e.g. [1], p. 222, 225, [2], p. 642 and [3]) that some beating artefacts (pseudo-moirés) may appear in the sampled signal *g*(*x*_*k*_) when *g*(*x*) is sampled with a sampling frequency *f*_s_ which *does* respect the Nyquist condition, but is still very close to the Nyquist limit itself (figure 1*b*).

Although these beating effects are not really new, they have rarely been treated in the literature, and they still remain poorly understood. They are sometimes considered as reconstruction artefacts, but no deeper analysis is provided to explain their nature and properties. And sometimes they are even simply dismissed as ‘borderline artefacts’ that occur when we get too close to the limits of the sampling theorem. However, it turns out that such beating effects are not limited to cases bordering on the Nyquist condition [4]. On the contrary, similar phenomena may also appear in many other cases that are by far *within* the Nyquist range. This happens, for example, when sampling a cosine signal *g*(*x*)=cos(2*πfx*) whose frequency *f* is close to (1/3)*f*_s_ or (1/4)*f*_s_, i.e. far below half of the sampling frequency. These beating artefacts are clearly not aliasing effects, as they appear where the Nyquist condition is fully satisfied. As we will see in greater detail in §3, these beating artefacts also have several other intriguing properties. In particular, their periods (or frequencies) are not represented in the Fourier spectrum, although they are clearly visible in the sampled signal. But although these beating artefacts are not aliasing or moiré effects, in many ways their behaviour is very similar to that of true sampling moirés. This explains, indeed, why they are often called in the literature ‘pseudo-moirés’. We will call these artefacts hereinafter *sub-Nyquist artefacts*.

The fact that these moiré-like artefacts are not visible by our main moiré investigation tool, the Fourier theory, makes them more difficult to analyse, but therefore also more interesting and challenging. In this paper, we address this challenge, and show how we can investigate these phenomena and incorporate them, together with the classical sampling moiré effects, within a common unified framework, based on sampling theory and on the moiré theory.

Our work is structured as follows: we start in §3 with an overview of the basic notions and terms that will be used in the sequel. (Readers who already master parts of this material may simply skip the paragraphs in question.) In §4, we provide the initial setting, and illustrate the nature of the phenomena in question by means of some typical examples, using the simple case of the cosine signal *g*(*x*)=*cos*(2*πfx*). Then, in §5, we derive a fundamental theorem which clearly explains the mathematical nature of the sub-Nyquist artefacts, and we show that classical sampling moiré effects are in fact a particular case of this theorem. Thus, by treating both classical sampling moirés and sub-Nyquist artefacts with the same unified tools, we put them all under the same roof. Our theorem as presented in §5 is limited to the particular case of the cosine signal, but then, in §6, we extend it to any general periodic signals. In §7, we discuss the nature of the sub-Nyquist artefacts as reconstruction artefacts. In §8, we discuss the potential impact of sub-Nyquist artefacts in signal-processing applications. And finally, our conclusions are presented in §9. Several appendices are provided in the electronic supplementary material.

The material presented in this paper is situated on the border between sampling theory and the moiré theory, and it benefits from both points of view, which are often dual and complementary. Because this paper basically aims at a signal-processing audience, which is not necessarily acquainted with the moiré theory, references are given whenever using concepts from the moiré theory. In addition, for the sake of completeness, electronic supplementary material, Appendix A provides a brief review of the main moiré-theory notions and terms that are being used here.

In addition to the figures which illustrate the discussions in the paper itself, two interactive Mathematica^®^ applications are also provided in the electronic supplementary material, along with their user's guide. Readers are encouraged to use these applications while reading this paper in order to better examine the cases under discussion, and for experimenting with any other cases they may wish to test. By manipulating the different parameters one may obtain a vivid graphic demonstration of the various sampling artefacts in question and of their dynamic behaviour, both in the signal domain and in the Fourier spectral domain. The source programs of these applications (which were also used to generate the figures of this paper) are provided, too. In addition, a glossary of the main terms which make use of these applications for illustrating the various terms is also provided in the electronic supplementary material.

## 3. Background and basic notions

Let *g*(*x*) be a continuous signal, and let *f*_max_ denote its maximum frequency (finite or infinite, depending on *g*(*x*)). As we know from the classical sampling theory, when *g*(*x*) is sampled at a frequency *f*_s_ which does not satisfy the Nyquist criterion, i.e. when *f*_s_<2*f*_max_ (and hence *f*_max_>(1/2)*f*_s_), the signal's frequencies above (1/2)*f*_s_ (and below −(1/2)*f*_s_) fold-over into the frequency range −(1/2)*f*_s_…(1/2)*f*_s_. In other words, the resulting sampled signal *g*(*x*_*k*_) contains new false frequencies within this range, which do not exist in the original signal *g*(*x*). This phenomenon is known as *aliasing*.

### 3.1 Sampling moiré effects

Aliasing artefacts may occur in the sampled signal whether *g*(*x*) is periodic or not. But when *g*(*x*) is periodic, so that its spectrum is impulsive, aliasing artefacts may become even more spectacular, due to their periodicity. From the point of view of the moiré theory (see the electronic supplementary material, Appendix A), the new folded-over low frequencies that we may get when sampling a periodic signal *g*(*x*) correspond to a *sampling moiré effect*.

**Figure 2.**
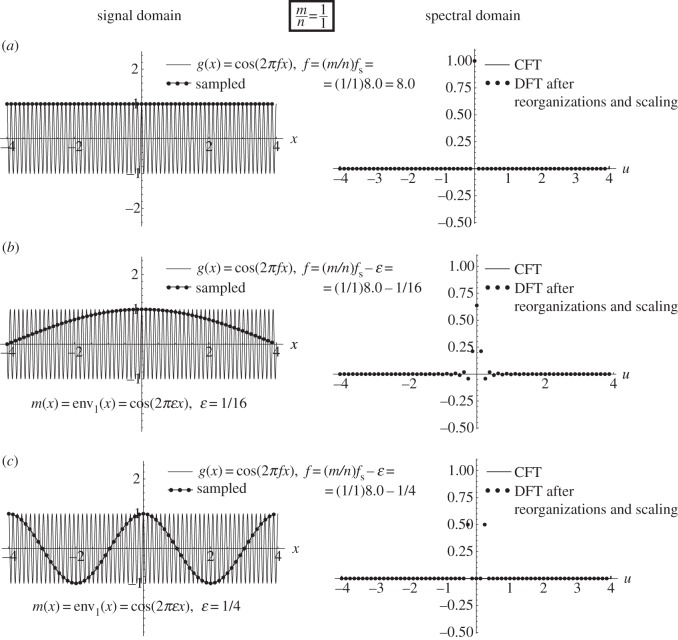
The artefact that occurs when *f*≈(1/1)*f*_s_ is a true first-order sampling moiré (figure 1*c*). Each row shows in the left-hand panel the periodic signal *g*(*x*)=*cos*(2*πfx*) having frequency *f*, as well as its sampled version after being sampled with a sampling frequency of *f*_s_=8 (i.e. with a sampling interval of Δ*x*=1/*f*_s_=1/8). The right-hand panels show the respective CFT of the continuous signal *g*(*x*), along with the DFT of its sampled version (after having applied the required reorganizations and scalings). The only difference between the three rows is in the frequency *f* of the original signal *g*(*x*): (*a*) *f*=*f*_s_ (the singular state), (*b*) *f*=*f*_s_−1/16 and (*c*) *f*=*f*_s_−1/4. The highly visible (1/1)-order artefact is generated because the sampled points *g*(*x*_*k*_) fall along a single low-frequency curve, which is simply a stretched version of *g*(*x*).

A sampling moiré effect is generated due to the interaction between the given periodic signal *g*(*x*) and the periodic sampling process. It consists of a new false low-frequency *f*_M_ which appears in the sampled signal *g*(*x*_*k*_) and in its spectrum, although it does not exist in the original signal *g*(*x*). For example, suppose that *g*(*x*) is the cosine signal cos(2*πfx*), whose spectrum consists of a single impulse pair located at *f* and −*f*. When the frequency *f* of our given signal is close to *f*_s_, i.e. when *f*_s_−*f*≈0, aliasing occurs, and the folded-over frequency falls at the point *f*_M_=*f*_s_−*f*, i.e. very close to the spectrum origin. The new false low-frequency *f*_M_ we therefore see in the sampled signal is a sampling moiré effect. This sampling moiré is shown in figure 2 for some values of *f* close to *f*_s_ (note that in all our figures we always use *f*_s_=8). As a second example, suppose that the frequency *f* of our given cosine signal is close to 2*f*_s_, i.e. that 2*f*_s_−*f*≈0. Here, too, a false low frequency appears in the sampled signal, but this time due to an interaction between *f* and 2*f*_s_. The folded-over frequency in this case is *f*_M_=2*f*_s_−*f*, which is again a very low frequency, located close to the spectrum origin. This effect is called a second-order sampling moiré.

Similarly, a sampling moiré effect occurs in the sampled signal whenever *f* approaches an integer multiple of *f*_s_, i.e. whenever *mf*_s_−*f*≈0, *m*=1,2,…. In each of these cases, a new low-frequency *f*_M_=*mf*_s_−*f* is generated in the spectrum of the sampled signal, close to the spectrum origin. Note that a sampling moiré effect is clearly visible both in the signal domain (as a false low-frequency signal) and in the spectral domain (as a folded-over false low-frequency spike), like in figure 2. This is a key characteristic property of the moiré effects.

### 3.2 Sub-Nyquist artefacts

**Figure 3.**
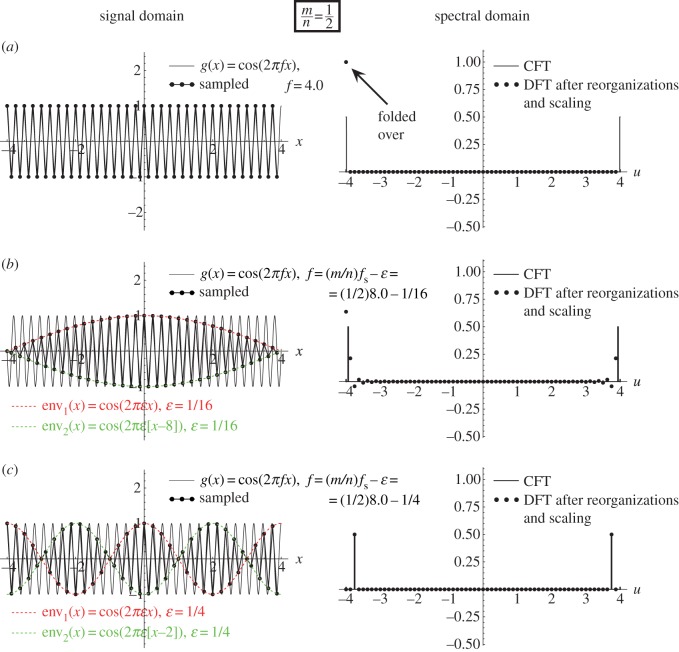
The (1/2)-order sub-Nyquist artefact; see figure 1*b*. This figure is similar to figure 2, except for the signal-frequency *f* being used in each row: (*a*) *f*=(1/2)*f*_s_ (the singular state), (*b*) *f*=(1/2)*f*_s_−1/16 and (*c*) *f*=(1/2)*f*_s_−1/4. The highly visible (1/2)-order sub-Nyquist artefact is generated because consecutive points *g*(*x*_*k*_) of the sampled signal alternately jump from one of the *n*=2 modulating envelopes to the other (each of the two modulating envelopes being simply a stretched and shifted version of *g*(*x*)). These two interlaced modulating envelopes are highlighted in the figure in different colours.

**Figure 4.**
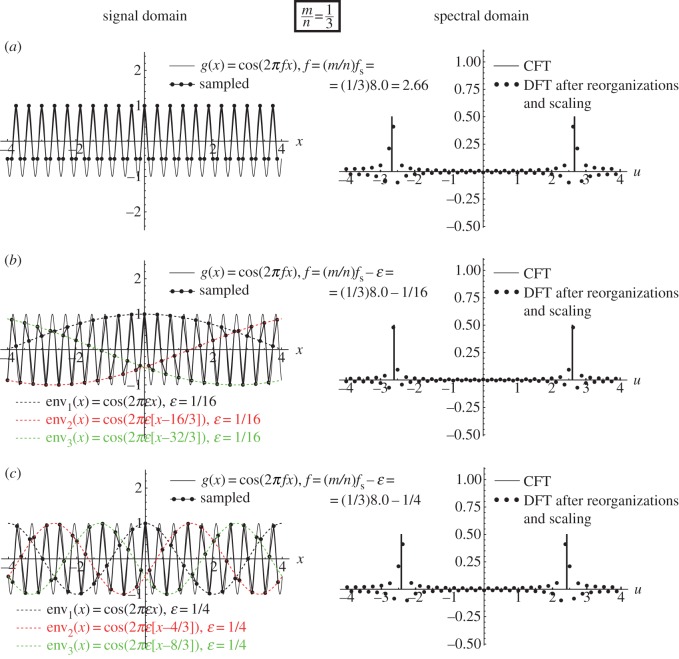
The (1/3)-order sub-Nyquist artefact. This figure is similar to figures 2 and 3, except for the signal-frequency *f* being used in each row: (*a*) *f*=(1/3)*f*_s_ (the singular state), (*b*) *f*=(1/3)*f*_s_−1/16 and (*c*) *f*=(1/3)*f*_s_−1/4. The highly visible (1/3)-order sub-Nyquist artefact is generated because consecutive points *g*(*x*_*k*_) of the sampled signal alternately jump from one of the *n*=3 modulating envelopes to the others (each of the three modulating envelopes being simply a stretched and shifted version of *g*(*x*)). These three interlaced modulating envelopes are highlighted in the figure in different colours.

As we have seen above, a sampling moiré effect is generated when the frequencies *f* and *f*_s_ satisfy *f*≈*mf*_s_ with an integer *m*. However, it turns out more generally that sampling artefacts may occur whenever *f*≈(*m*/*n*)*f*_s_ with integer *m*, *n*.^2^ When *n*=1, the artefacts we obtain are simply sampling moiré effects. But when *n*≠1 a different type of artefacts occurs in the sampled signal. These are precisely the *beating artefacts* on which we focus in the present work. As we can see in figures 3 and 4, these beating artefacts are intriguing for several reasons.

(i) They may appear where the Nyquist condition is fully satisfied, so that no aliasing or sampling moiré artefacts should be present. For example, in figure 4 the frequency of the original function *g*(*x*)=*cos*(2*πfx*) is close to (1/3)*f*_s_, i.e. far below half of the sampling frequency.
(ii) Unlike in aliasing or moiré phenomena, the periods (or frequencies) of these beating artefacts are not represented in the Fourier spectrum, although they are clearly visible in the sampled signal. To see this, compare the spectra of our beating artefacts in any of figures 3 and 4 with the spectra of the sampling moiré effects of figure 2: while in figure 2 the low frequency in the signal domain is also represented in the spectral domain as a folded-over frequency, in figures 3 and 4 the low-frequency structures in the signal domain *do not* appear in the spectrum.^3^
(iii) Furthermore, in the signal domain, the beating effect in question does not really correspond to a low-frequency signal, but rather to a highly oscillating signal that is only *modulated* by low-frequency envelopes (compare the beating effect in figures 3 and 4 with the true sampling moiré effect in figure 2). Note that this may provide a clue to the understanding of point (ii): the low frequencies in the signal domain belong to the *modulating envelopes* of the sampled signal, but they are not included in the sampled signal itself.

We call these beating effects *sub-Nyquist artefacts*, reflecting the fact that they may occur well below the Nyquist frequency. But as we will see later, these artefacts may also occur above the Nyquist frequency (see Remark 8 in electronic supplementary material, Appendix C).

Obviously, the first approach that comes to one's mind for investigating such phenomena is based on the Fourier theory, the main investigation tool being used in the moiré theory and in signal processing, but the fact that our beating artefacts are not visible in the spectrum of the sampled signal (see point (ii) above) makes them more difficult to analyse.

In order to explore these ‘hidden’ cases, we propose the following approach: we let the frequency *f* of the continuous signal *g*(*x*) sweep along the frequency axis, just as we do while running from row to row in figure 1, and we see what happens when the signal-frequency *f* approaches critical points such as multiples or integer fractions of the sampling frequency *f*_s_. Some typical cases will be described in §4, in order to develop a better understanding of these phenomena and of their properties. Based on the insights gained in §4, we will proceed in §§5 and 6 to the mathematical analysis of these phenomena. For the sake of simplicity, we start our exploration using the simple case of the cosine function; later on we will generalize our results to any general periodic signals.

## 4. Sampling a continuous cosinusoidal signal

Suppose we are given the continuous signal *g*(*x*)=*cos*(2*πfx*), i.e. a cosine function with frequency *f*, and that we sample it using the sampling frequency *f*_s_. We denote the resulting sampled signal by *g*(*x*_*k*_)=*cos*(2*πfx*_*k*_), where *x*_*k*_=*k*Δ*x*, and Δ*x*=1/*f*_s_ is the sampling step. How does the sampled signal behave when we gradually vary the cosine frequency *f* or the sampling frequency *f*_s_? Obviously, both approaches (varying *f* or varying *f*_s_) are in fact equivalent, but for the sake of simplicity (technical considerations when applying discrete Fourier transform (DFT) to our sampled signal), we prefer to keep the sampling frequency *f*_s_ fixed, and to study what happens when we gradually vary the frequency *f* of our given continuous function (as in figure 1). Note that in all our figures we always use the fixed sampling frequency *f*_s_=8; this choice is convenient for the DFT being used in our figures, but in principle, any other *f*_s_ value could also be chosen, be it integer or not.

Let us now start varying the frequency *f* along the frequency axis, and stop to explore a few typical examples of artefacts that we encounter on our way. Interested readers may do this exercise on their own using the provided interactive applications, and dynamically watch the various artefacts as they gradually appear and disappear while *f* is slowly being varied.^4^

**Example 4.1 (sampling moiré effect)**

Let us first explore what happens in the frequency range around *f*≈*f*_s_. This is shown in figure 1*c*, and in greater detail in figure 2. In this case, we have

4.1 $$f=f_{s}-\varepsilon ,$$

where *ε* denotes a small positive value.^5^ As this case does not respect the Nyquist condition *f*≤(1/2)*f*_s_ (or *f*_s_≥2*f*) required by the sampling theorem, aliasing occurs here. Indeed, looking at figure 2 we see that our sampled points fall on a low-frequency cosinusoidal curve whose frequency is precisely *ε*:

4.2 m(x)=cos(2πεx).

This is, in fact, a one-dimensional sampling moiré effect, which simply obeys the basic rules of the moiré theory (e.g. [6], §4.2):

(i) its frequency is *ε*=*f*_s_−*f*; and
(ii) its intensity profile *m*(*x*) is an expanded version of the original continuous function *g*(*x*), that is simply stretched (or magnified) along the *x* axis by an expansion (magnification) rate of *f*/*ε*, in order to satisfy (i):^6^
  $$m(x)=g\left(\frac{\varepsilon }{f}x\right).$$

As we can see in figure 2*a*, when *f* precisely equals *f*_s_ (i.e. when *ε*=0) we get the *singular state* of the sampling moiré effect, where the moiré period 1/*ε* is infinitely large and therefore invisible. But when *f* slightly moves away from the frequency *f*_s_ (to either direction) the moiré ‘comes back from infinity’, as shown in figure 2*b*, and becomes again visible with a long period 1/*ε* (small frequency *ε*). As *f* moves farther away from the singular state *f*=*f*_s_, i.e. as |*ε*| gradually increases (see figure 2*c*), the period of the moiré effect becomes smaller and smaller, until it can no longer be detected.^7^

Note that in terms of the sampling theory, this moiré effect is simply a fold-over artefact due to aliasing (see, for instance, example 5.4 in [7], §5.3).

Similar results are also obtained when we let *f* explore the frequency range around *f*≈*mf*_s_ for any integer *m*>1, giving an *m*-th-order sampling moiré. And indeed, when

4.3 $$f=mf_{s}-\varepsilon ,$$

our sampled points *g*(*x*_*k*_) will reside, once again, on the same low-frequency cosinusoidal curve (3.2) having the frequency *ε*.

So far we have seen what happens when the frequency *f* of our continuous cosine *g*(*x*) approaches integer multiples of the sampling frequency *f*_s_. Let us now continue varying the frequency *f*, and study what happens when we arrive to fractional multiples of *f*_s_.

**Example 4.2 (sub-Nyquist artefact)**

Let us focus on the frequency range around *f*≈(1/2)*f*_s_ (i.e. 2*f*≈*f*_s_), as shown in figure 1*b* or in greater detail in figure 3, i.e. when

4.4 $$f=\frac{1}{2}f_{s}-\varepsilon .$$

The above equation implies that we are located here in the ‘safe’ side of the Nyquist frequency (1/2)*f*_s_, meaning that the sampling condition stipulated by the sampling theorem, *f*≤(1/2)*f*_s_ (or *f*_s_≥2*f*), is fully satisfied, and no aliasing or moiré effects should be present. Yet, looking at figure 3, we see that a low-frequency beating effect is visible in the sampled signal, due to a low-frequency cosinusoidal modulation. At the singular frequency itself, *f*=(1/2)*f*_s_ (figure 3*a*), the modulating envelope of the beating effect has an infinitely long period, and is therefore invisible, but as *f* moves away from this singular frequency, i.e. as |*ε*| gradually increases (figure 3*b*,*c*), the period of the beating effect becomes smaller and smaller, until it finally disappears. As we have seen above, this behaviour is typical to a moiré effect. However, our present beating effect is not a true sampling moiré but rather a *pseudo*-moiré effect [8], or using our present terminology, a sub-Nyquist artefact: as we can see in figure 3, in our case no corresponding low-frequency impulses exist in the respective spectra (compare the low-frequency zone around the origin in the DFT spectra of figure 3 with the same zone in figure 2, where a true aliased sampling moiré does exist). Furthermore, even in terms of the signal domain, the beating effect in question does not really represent a low-frequency signal (as in figure 2), but rather a highly oscillating signal that is only *modulated* by two interlaced low-frequency cosinusoidal envelopes. These two envelopes, which are highlighted in figure 3 by different colours, have the envelope frequency *ε*=(1/2)*f*_s_−*f*. Because the frequency *ε* is much lower than the frequency *f* of the original cosine function *g*(*x*) being sampled, this artefact may become quite conspicuous and distort our perception of the true nature of the original signal.

The beating effect we have described in example 4.2 occurs when the frequency *f* of the cosine function being sampled is located slightly below half of the sampling frequency, i.e. almost at the border of the ‘safe’ frequency zone stipulated by the sampling theorem. This case could be therefore dismissed as a ‘borderline’ artefact that occurs when we get too close to the limits of the theorem. However, it turns out that such sub-Nyquist artefacts may also appear when the cosine frequency *f* is well below (1/2)*f*_s_, as clearly illustrated by the following example.

**Example 4.3 (sub-Nyquist artefact)**

Consider figure 4, which shows what happens when we arrive at the frequency range around *f*≈(1/3)*f*_s_ (i.e. 3*f*≈*f*_s_), and in particular when

4.5 $$f=\frac{1}{3}f_{s}-\varepsilon .$$

Figure 4*a* shows the situation when the cosine frequency is exactly *f*=(1/3)*f*_s_=2.66, and figure 4*b*,*c* shows what happens when the cosine frequency *f* gradually moves away from this singular frequency, i.e. when |*ε*| is gradually increased. As we can see, the resulting visual effect in the signal domain resembles the modulation effect we obtained in figure 3, where the cosine frequency *f* was close to (1/2)*f*_s_=4, except that in figure 4 the beating effect corresponds to *three* interlaced cosinusoidal envelopes rather than two. The three interlaced envelopes obtained in this case, which are highlighted in figure 4 by different colours, have the envelope frequency *ε*=(1/3)*f*_s_−*f*. Note that in this case, too, as the frequency *ε* is much lower than the frequency *f* of the original signal *g*(*x*) being sampled, this artefact may become quite conspicuous. But once again, this beating effect in the sampled signal is misleading, as it is only a sampling artefact and it does not reflect the true behaviour of our original continuous-world signal *g*(*x*). Furthermore, its low frequency is not represented in the DFT spectra, meaning that it is not a true sampling moiré effect.

Pursuing our sweep along the frequency axis, it turns out that similar sub-Nyquist artefacts may occur in many other cases too, even well below the Nyquist frequency limit set-up by the sampling theorem. In general, sampling artefacts occur whenever our cosine frequency *f* is located around a rational fraction *m*/*n* of the sampling frequency *f*_s_, i.e. whenever *f*≈(*m*/*n*)*f*_s_. As we have seen above, when *n*=1 the resulting artefact is simply a sampling moiré effect. But when *n*>1 we obtain a beating artefact that is not a moiré effect but rather a sub-Nyquist artefact; we denote it as a (*m*/*n*)-*order* *sub-Nyquist* *artefact*. This artefact manifests itself in a curious way—it forces all the sampled points *g*(*x*_*k*_) of our given continuous signal *g*(*x*) to fall on *n* interlaced modulating envelopes:

4.6 $$\begin{array}{l} {\text{env}}_{1}(x)=\text{cos(}2\pi \varepsilon x),\\ {\text{env}}_{2}(x)=\text{cos(}2\pi \varepsilon [x+a]),\\ \cdots \\ {\text{env}}_{n}(x)=\text{cos}(2\pi \varepsilon [x+(n-1)a]), \end{array}\}$$

where the frequency of each envelope is *ε*=(*m*/*n*)*f*_s_−*f*, and the shift *a* between adjacent envelopes equals *m*/*n*-th of the cosinusoidal envelope's period 1/*ε*, i.e. *a*=*m*/(*nε*). We will see the formal derivation of these expressions in §5.

Interestingly, the true moiré cases (like in figure 2) also obey the very same rule, but this time with *n*=1 envelopes, so that no interlacing occurs. This confirms, once again, the common nature of sampling moiré effects and sub-Nyquist artefacts.

Obviously, the most interesting (*m*/*n*)-order sub-Nyquist artefacts are those with *m*<*n*/2, for which (*m*/*n*)*f*_s_ is lower than (1/2)*f*_s_, i.e. below half of the sampling frequency (like the (1/3)-order, (2/5)-order, etc.), but other cases such as the (2/3)-order, (3/2)-order, (3/5)-order, etc., may also be considered; see Remark 8 in the electronic supplementary material, Appendix C.

So how can we explain these artefacts that are ‘hidden’ to the Fourier spectra? Why do these visible phenomena occur even when *f* is located in the ‘safe’ side of the Nyquist frequency (1/2)*f*_s_, meaning that the sampling condition stipulated by the sampling theorem, *f*≤(1/2)*f*_s_, is fully satisfied? We will see the answer in the following sections, first for the particular case of the cosine function *g*(*x*)=cos(2*πfx*), in §5, and then for the general case with any periodic function *g*(*x*), in §6.

## 5. Derivation of the interlaced modulation envelopes

Let us first explain the phenomena which occur in the (*m*/*n*)-order sub-Nyquist artefact in the simplest case, when we are sampling a cosine function *g*(*x*) with frequency *f* and period *p*=1/*f*:

5.1 g(x)=cos(2πfx).

When we sample *g*(*x*) at the sampling frequency *f*_s_, i.e. using a sampling step of Δ*x*=1/*f*_s_, we obtain the sampled signal:

5.2 $$g(x_{k})=\text{cos}(2\pi fx_{k})$$

with *x*_*k*_=*k*Δ*x*=*k*/*f*_s_.

Suppose, first, that the frequency of the original continuous cosine *g*(*x*) is exactly

5.3 $$f=\frac{m}{n}f_{s}.$$

In this case *p*=(*n*/*m*)Δ*x*, meaning that we have exactly *n*/*m* samples in each period *p* of the given cosine, and the sampling step Δ*x* is *m*/*n* of the cosine period *p*. The sampled signal we obtain in this case is

5.4 $$g(x_{k})=\text{cos}(2\pi fx_{k})=\text{cos}\left(2\pi \left[\frac{m}{n}f_{s}\right]\frac{k}{f_{s}}\right)=\text{cos}\left(2\pi k\frac{m}{n}\right).$$

This corresponds, indeed, to the singular state of the (*m*/*n*)-order sub-Nyquist artefact, which is systematically plotted in figures 2–4 in row (*a*).

Now, suppose that the frequency of the given cosine *g*(*x*) is not exactly *f*=(*m*/*n*)*f*_s_, but rather

5.5 $$f=\frac{m}{n}f_{s}+\varepsilon ,$$

where *ε* may be either negative, as we did so far, or positive. The sampling frequency *f*_s_ remains unchanged, so that we still have *x*_*k*_=*k*Δ*x*=*k*/*f*_s_. The sampled signal is, in this case

5.6 g(xk)=cos(2πfxk)=cos(2π[mnfs+ε]kfs)g(xk)=cos(2πkmn+2πεkfs)g(xk)=cos(2πxkε+2πϕ)with ϕ=kmng(xk)=cos(2πε[xk+ϕε]).}

As we can see, this is a sampled cosine having frequency *ε* (i.e. period 1/*ε*) and a nominal shift of *ϕ*/*ε*, i.e. a *relative shift* of *ϕ* periods (see the definition of the various phase-related terms and notations in the electronic supplementary material, Appendix B).

This means that:

(1) for integer *ϕ*=*k*(*m*/*n*), namely for *k*=0, *n*, 2*n*, 3*n*,… we have
  $$g(x_{k})=\text{cos}(2\pi x_{k}\varepsilon +0)=\text{cos}(2\pi \varepsilon [x_{k}+0]).$$
  This cosine has the phase *φ*=0, i.e. a shift of 0 or of an integer multiple of its period 1/*ε*.
(2) For *k*=1, *n*+1, 2*n*+1, 3*n*+1,… we have
  $$g(x_{k})=\text{cos}\left(2\pi x_{k}\varepsilon +\frac{m}{n}2\pi \right)=\text{cos}\left(2\pi \varepsilon \left[x_{k}+\frac{m}{n}\frac{1}{\varepsilon }\right]\right).$$
  This cosine has the phase *φ*=(*m*/*n*)2*π* , i.e. a shift of *m*/*n* times the period 1/*ε*.
(3) For *k*=2, *n*+2, 2*n*+2, 3*n*+2,… we have
  $$g(x_{k})=\text{cos}\left(2\pi x_{k}\varepsilon +2\frac{m}{n}2\pi \right)=\text{cos}\left(2\pi \varepsilon \left[x_{k}+2\frac{m}{n}\frac{1}{\varepsilon }\right]\right).$$
  This cosine has the phase *φ*=2(*m*/*n*) 2*π*, i.e. a shift of 2(*m*/*n*) times the period 1/*ε*.
  …
(*n*) For *k*=*n*−1, 2*n*−1, 3*n*−1, 4*n*−1,… we have
  $$g(x_{k})=\text{cos}\left(2\pi x_{k}\varepsilon +(n-1)\frac{m}{n}2\pi \right)=\text{cos}\left(2\pi \varepsilon \left[x_{k}+(n-1)\frac{m}{n}\frac{1}{\varepsilon }\right]\right).$$
  This cosine has the phase *φ*=(*n*−1)(*m*/*n*) 2*π*, i.e. a shift of (*n*−1)(*m*/*n*) times the period 1/*ε*.

This means that the successive sampled points of our original cosine function, *g*(*x*_*k*_)=*cos*(2*πfx*_*k*_), *k*=0,1,2,… fall intermittently on one of *n* cosinusoidal curves (that we call *envelopes*), which have all the same frequency *ε* and period 1/*ε*, and which only differ from each other in their phase. More precisely, these *n* envelopes only differ from each other by successive relative shifts of *m*/*n* periods 1/*ε*, i.e. by successive shifts of *a*=*m*/(*nε*). This corresponds, indeed, to the situation in figures 2*b*,*c*, 3*b*,*c* and 4*b*,*c*.

We have proved, therefore, the following result.

**Theorem 5.1**

*Suppose we are given a continuous cosine function g(x)=cos(2πfx) having frequency f and period p=1/f, and that we sample this function at the sampling frequency f*_s_*, i.e. with a sampling step of* Δ*x*=1/*f*_s_*. If the frequency f of our given cosine g(x) differs by ε from the singular frequency m/n f*_s_ *for some integers m and n:*

$$f=\frac{m}{n}f_{s}+\varepsilon ,$$

*(where ε may be positive or negative), then the successive sampled points of our original cosine function, g(x*_*k*_*)=cos(2πfx*_*k*_*), k=0,1,2,… fall intermittently on one of n interlaced cosinusoidal envelopes, which have all the same frequency f*_env_*=ε and period p*_env_*=1/ε, and which only differ from each other in their phase. Any two successive envelopes are simply displaced from each other by m/n of their period p*_env_*, i.e. by a shift of a=m/(nε).*

This theorem explains the (*m*/*n*)-order sub-Nyquist artefacts in the case of a cosinusoidal function. It is interesting to note that this theorem remains valid even in cases with *n*=1. In such cases, all the sampled points *g*(*x*_*k*_) fall on a single envelope, which corresponds to a *true* moiré effect: there are no interlaced envelopes, and the sampled points no longer jump intermittently from one curve to another as they do in a pseudo-moiré effect.

## 6. Sub-Nyquist artefacts in general periodic functions

In the previous sections, we have only considered sub-Nyquist artefacts which occur when sampling a cosinusoidal function.

However, the same results can be also demonstrated, exactly in the same manner, for *any* periodic function *g*(*x*), by considering the Fourier series development of *g*(*x*). The detailed demonstration is provided in the electronic supplementary material, Appendix D. We thus obtain the following generalization of theorem 5.1.^8^

**Theorem 6.1**

*Suppose we are given a continuous periodic function g(x) having frequency f and period p=1/f, and that we sample this function at the sampling frequency f*_s_*, i.e. with a sampling step of* Δ*x*=1/*f*_s_*. If the frequency f of our given function g(x) differs by ε from the singular frequency (m/n)f*_s_ *for some integers m and n:*

$$f=\frac{m}{n}f_{s}+\varepsilon ,$$

*(where ε may be positive or negative), then the successive sampled points of our original function, g(x*_*k*_*), k=0,1,2,… fall intermittently on one of n interlaced low-frequency envelopes, which are simply expanded (stretched) versions of g(x) having the frequency f*_env_*=ε and period p*_env_*=1/ε, and which only differ from each other in their phase. Any two successive envelopes are displaced from each other by m/n of their period p*_env_*, i.e. by a shift of a=m/(nε).*

Once again, this theorem remains valid in cases with *n*=1, too; in such cases all the sampled points *g*(*x*_*k*_) fall on a single envelope, which corresponds to a *true* moiré effect.

**Figure 5.**
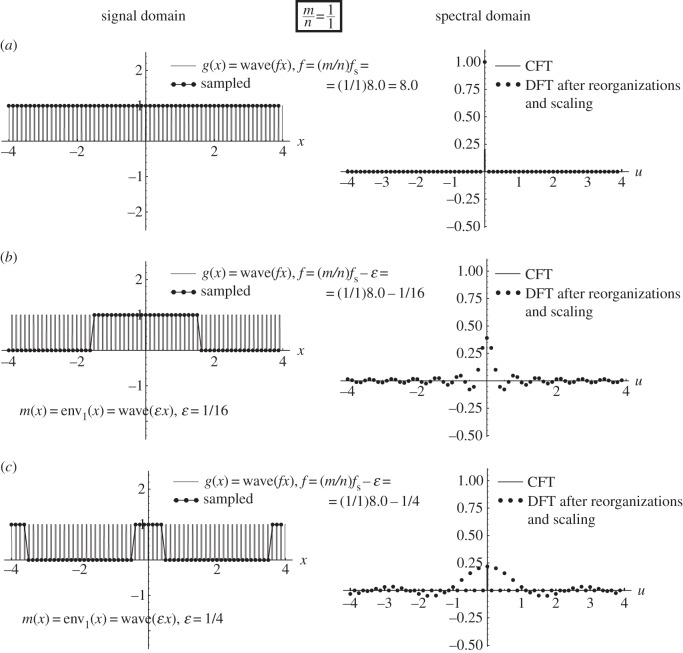
(*a*–*c*) This figure is similar to figure 2, except that the original continuous function being sampled is the periodic square wave *g*(*x*)=*wave*(*fx*) with frequency *f* (and an opening ratio of *τ*/*p*=1/5, meaning that the 1-valued part of the wave occupies 1/5 of its period *p*=1/*f*). The highly visible (1/1)-order artefact is generated because the sampled points *g*(*x*_*k*_) fall along a single low-frequency curve, which is simply a stretched version of *g*(*x*).

**Figure 6.**
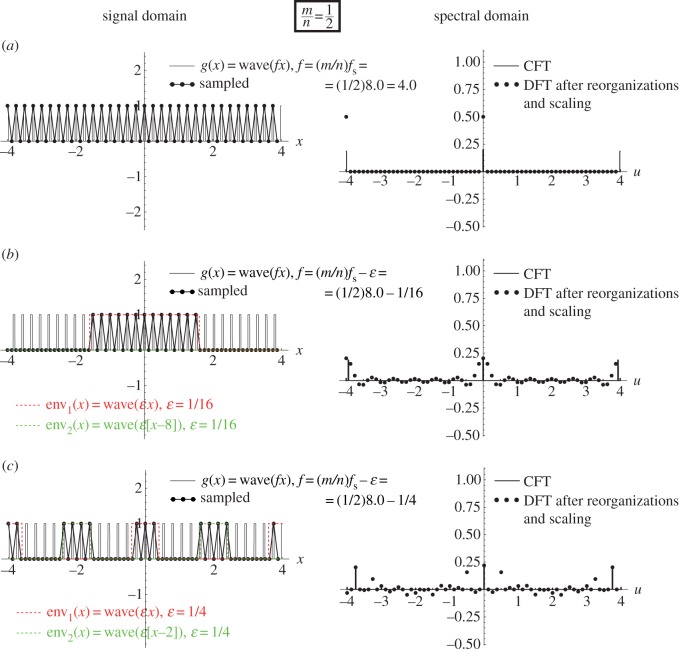
(*a*–*c*) This figure is similar to figure 3, except that the original continuous function being sampled is the periodic square wave *g*(*x*)=*wave*(*fx*) (with frequency *f* and an opening ratio of *τ*/*p*=1/5). The highly visible (1/2)-order sub-Nyquist artefact is generated because consecutive points *g*(*x*_*k*_) of the sampled signal alternately jump from one of the *n*=2 modulating envelopes to the other (each of the two modulating envelopes being simply a stretched and shifted version of *g*(*x*)). These two interlaced modulating envelopes are highlighted in the figure in different colours.

To illustrate this generalized theorem graphically, consider figures 5 and 6. These figures are similar to figures 2 and 3, except that the original continuous function being sampled here is a square wave with frequency *f* rather than a cosine function with frequency *f*. In figure 5 (just like in its cosine counterpart figure 2), which corresponds to the (1/1)-order case, the sampled points *g*(*x*_*k*_) fall on exactly one envelope. However, in figure 6 (just like in figure 2, its cosine counterpart), which corresponds to the (1/2)-order case, the sampled points *g*(*x*_*k*_) fall on two interlaced envelopes, that are highlighted in our figure by two different colours. In all figures 2–6, each of the envelopes we obtain is simply an expanded (stretched) version of the original continuous function *g*(*x*) being sampled, having the frequency *ε* (and period 1/*ε*) rather than *f* (and *p*=1/*f*) as in the original function *g*(*x*) itself. The magnification rate is, therefore, (1/*ε*)/(1/*f*)=*f*/*ε* along the *x* axis. Furthermore, whenever *n*>1 so that interlacing does occur, successive interlaced envelopes are displaced from each other by *m*/*n* of their period 1/*ε*, i.e. by a shift of *a*=*m*/*nε*.^9^

Finally, it should be noted that theorem 6.1 only holds when we are sampling a *periodic* function *g*(*x*). Nevertheless, this theorem may be of interest even when the function *g*(*x*) is aperiodic but still contains a strong periodic component (which manifests itself as a spike in the spectrum of *g*(*x*)).

## 7. Discussion: sampling and reconstruction considerations

As we have mentioned in §2, although sub-Nyquist artefacts are generated during the sampling process, they are often considered as reconstruction artefacts (meaning that they appear as a result of *poor reconstruction*). Let us explain this in some more detail.

Sampling is the process that converts a continuous signal to a discrete one, while reconstruction is the process that recreates a continuous signal from its samples ([2], §14.10.5, [1]). Note that theoretically, a sampled signal consists of zero-width impulses (of varying strengths), and it is precisely the reconstruction process that brings back the ‘flesh’ around each of the sampled ‘bones’. Now, according to the classical sampling theorem (see, e.g. [9], §5.4, [10], §8–7, [11], p. 115 or [12], p. 1593), all the information in the original continuous signal *g*(*x*) is preserved in its sampled version *g*(*x*_*k*_), if the sampling frequency is at least twice the highest frequency contained in *g*(*x*). Under this condition, the theorem guarantees that the original continuous signal can be perfectly reconstructed by sinc interpolation, i.e. by convolving the sampled signal *g*(*x*_*k*_) with a specified narrow sinc function (that is precisely the inverse Fourier transform of the rectangular window function in the spectral domain that cuts off all the frequencies beyond half of the sampling frequency; see [9], p. 83). This result remains true even if the sampling process itself introduces into the sampled signal *g*(*x*_*k*_) new modulation or beating effects (sub-Nyquist artefacts) that did not exist in the original continuous signal *g*(*x*), as it happens, for instance, in figure 3. In other words, if reconstruction is done as stipulated by the sampling theorem, the theorem indeed guarantees that these artefacts will disappear in the reconstruction process.

However, in practice, the reconstruction of a sampled signal can never be done by a sinc interpolation as stipulated by the sampling theorem, because the sinc function extends ad infinitum to both directions. Instead, reconstruction is very often performed by means of a linear interpolation, meaning that successive sample points are simply connected by straight line segments, just as on the display of an oscilloscope. This is, indeed, what we are also doing in our figures. Now, because the reconstruction process is not performed as stipulated by the sampling theorem, it is clear that the original continuous signal *g*(*x*) cannot be perfectly reconstructed from the sampled signal *g*(*x*_*k*_). Indeed, if perfect reconstruction by sinc interpolation could be performed, the sub-Nyquist artefacts would have been eliminated in the reconstruction (interpolation) process, and we would obtain a continuous signal that is perfectly identical to *g*(*x*). However, when the reconstruction is performed by a non-ideal reconstruction method such as linear interpolation, the sub-Nyquist artefacts are not eliminated during the reconstruction process, and they remain visible in our resulting signal. Obviously, the closer we approach sinc interpolation the better are the results, but in the real world we can never reach the ideal conditions set by the sampling theorem.

Note, however, that true aliasing moiré effects, those that occur when the Nyquist condition is not satisfied, like in figure 2, would not be eliminated even by an ideal reconstruction: these artefacts, which *are* indeed represented by low frequencies in the spectrum, are embedded in the sampled signal and remain visible in the reconstructed signal too, whichever reconstruction method we may choose.

Having understood the origin and the nature of sub-Nyquist artefacts, an obvious question may now arise: as these artefacts cannot be eliminated in the reconstruction stage, is it possible to avoid them by judiciously choosing our sampling frequency? As one could guess, avoiding such ‘dangerous frequency zones’ is difficult, as they may lurk anywhere along the frequency axis, including in zones that fully respect the frequency condition required by the sampling theorem. A possible rule of thumb [4], p. 15 consists of taking *f*_s_>10*f* (i.e. *f*_s_≈(*n*/*m*)*f*>10*f* and hence *n*/*m*>10) rather than the Nyquist requirement of *f*_s_>2*f* (i.e. *f*_s_≈(*n*/*m*)*f*>2*f* and hence *n*/*m*>2). The reasoning behind this rule of thumb is that for high values of *n* the artefacts in question become rather negligible (see Remarks 2 and 4 in the electronic supplementary material, Appendix C). Nevertheless, a good practice when sampling a given signal having a strong periodic component of frequency *f* would be to avoid sampling-frequencies *f*_s_ in the close neighbourhood of (*n*/*m*)*f* for relatively small integers *n* and *m*. If these ‘dangerous zones’ are carefully avoided, one may choose even lower values of *f*_s_, which are often more practical to use. It should be mentioned that in some particular cases signals can be correctly reconstructed even when using a sampling rate much lower than the Nyquist requirement. This subject is being studied in the field of compressive sampling, which continues gaining interest since the work of Donoho [13] and Candès [14] first appeared in 2006. In the present contribution, we only address the general case, in which the classical sampling theorem applies, but possible extensions of this subject within other sampling frameworks could also be studied in future works.

## 8. Significance in applications

Sub-Nyquist artefacts may occur in various signal-processing applications, and one should be aware of their existence and of their correct interpretation. Each application involving analogue to digital conversion of continuous periodic signals, including simple sinusoidal waves, may give rise to such artefacts in the resulting digital signal, even if the original signal is band limited and the Nyquist condition is respected.

In many cases such phenomena (with *n*>1) are harmless, as they do not truly introduce new false frequencies into the system, as aliasing phenomena do. But in other cases a misunderstanding of these phenomena may lead to undesirable results even when *n*>1. This may happen, for example, in applications that rely on amplitude peak detection in the sampled signal. In such cases, false peaks may be detected in the sampled signal, even though they do not exist in the original continuous signal (e.g. figures 3 and 4).

Care should be taken in particular when the resulting signal has to undergo in subsequent processing steps any nonlinear operation (rectification, amplitude truncation, nonlinear amplification, etc.). A nonlinear operation may truly insert the envelope frequencies in question into the signal and its spectral representation [15], and hence flaw the processing results. This may happen, for example, when applying to the beating signal a nonlinear operation such as envelope detection; see also footnote 3 in §3.2. Other examples in the field of image processing include nonlinear operations such as quantization or halftoning [2], §13.1.2.

It should also be noted that when further processing of the sampled signal is required, one should always use the sampled signal itself, and not its reconstructed version (which is necessarily based on non-ideal reconstruction, as we have seen above). The reconstructed version of the sampled signal can be used for plotting or displaying purposes, but not for any further processing steps.

## 9. Conclusion

We show in this paper that sub-Nyquist artefacts and sampling moiré effects are, indeed, particular cases of the same phenomenon: whenever the frequency *f* of our given continuous periodic function *g*(*x*) is close to a critical point (*m*/*n*)*f*_s_ where *f*_s_ is the sampling frequency and *m* and *n* are integers, a visible artefact is generated in the resulting sampled signal. This artefact is explained by our main theorems: it occurs as, under these circumstances, the successive sampled points of our original function, *g*(*x*_*k*_), *k*=0,1,2,… fall intermittently on one of *n* interlaced low-frequency envelopes, which are simply expanded (stretched) versions of *g*(*x*) having the frequency *ε* and period 1/*ε*, and which only differ from each other in their phase. If *n*=1 then all the sampled points fall on a single envelope, and the resulting effect is simply a sampling moiré effect, which indeed behaves exactly as predicted in the moiré theory. However, when *n*>1, the successive sampling points *g*(*x*_*k*_) alternately jump from one modulating envelope to another, and the resulting effect is a sub-Nyquist artefact. If *g*(*x*) does not possess the *n*-th harmonic of its frequency *f*, as in the cosinusoidal case, this is a pure modulation phenomenon and it is not accompanied by a corresponding low-frequency impulse in the spectrum. Yet, as we have seen throughout this paper, in spite of this difference between true moiré effects and sub-Nyquist artefacts, they all behave in a very similar way. Let us mention, for example, their similar behaviour at the singular point or around it, their mirror-inversion depending on the sign of *ε* (i.e. depending on which side of the singular point they are located; see the electronic supplementary material, Remark 3 in Appendix C), etc. This striking similarity between true moirés and sub-Nyquist artefacts is indeed explained by the fact that they are both particular cases of the same phenomenon, as shown by our theorems.

Finally, as we have seen in §7, sub-Nyquist artefacts are really generated during the sampling process, but they remain visible due to imperfect reconstruction. Our analysis of the sub-Nyquist artefacts would be of interest even if these phenomena only existed in the sampled signal, and then disappeared during the reconstruction process, but because perfect reconstruction does not exist in the real world, so that sub-Nyquist artefacts cannot be completely eliminated from the reconstructed signal, the analysis provided here becomes all the more valuable and pertinent. Our results may be of interest to people working in a wide range of applications, where such artefacts may occur and should be therefore well understood.

## Supplementary Material

Appendies A-D of the main paper.

## Supplementary Material

Interactive Mathematica application.

## Supplementary Material

Interactive Mathematica application.

## Supplementary Material

Interactive Mathematica application.

## Supplementary Material

Interactive Mathematica application.

## Supplementary Material

User s guide for the interactive applications.

## Supplementary Material

Glossary of the main terms.

## Footnotes

1 Note that lower sampling frequencies may suffice for some special types of signals, but the classical sampling theorem addresses the general case.

2 Note that by *m*/*n* we always mean a *reduced* integer fraction. We will tacitly use this convention throughout this paper, even when it is not mentioned explicitly.

3 Of course, such frequencies can be made visible in the spectrum by applying to the beating signal in question a nonlinear operation such as envelope detection (rectification possibly followed by low-pass filtering [5], p. 216). This practical ‘trick’ can be used in applications to bypass the problem, but it cannot help us in the theoretical explanation of these phenomena.

4 To slow down the movement of the ‘*f*’ slider in the applications, hold down the Alt or Option key while dragging the slider with the mouse. The movement can be further slowed down by also holding down the Shift or Ctrl key, or both (see the user's guide of the applications in the electronic supplementary material).

5 The results obtained when taking *f*=*f*_s_+*ε* are similar, so we only need to consider one of the two cases; we will return to this point in greater detail in Remark 3 of electronic supplementary material, Appendix C.

6 According to a moiré theory result (e.g. [6], §4.2 or §10.9), the profile of this moiré is determined by the cyclic convolution of the profiles of the two periodic structures involved, the sampling comb III(*x*) and the original continuous function *g*(*x*), both being normalized to the same period. The profile of the resulting moiré is therefore equal to that of *g*(*x*), as a cyclic convolution of *g*(*x*) with an impulse comb gives back *g*(*x*). This resulting profile is then stretched according to point (i).

7 Theoretically, a moiré effect ‘exists’ even when its frequency gets far away from the spectrum origin; but practically, we may (and do) ignore it when it is no longer visible and pertinent.

8 Interestingly, the fact that *g*(*x*) may also contain higher harmonics of its basic frequency *f*, and that these harmonics may cause aliasing, does not affect the validity of the result. This point is explained by Remark 9 in the electronic supplementary material, Appendix C.

9 It is interesting to note that unlike the cosine function, the square wave is not band limited (its spectrum contains infinitely many harmonics of *f*). This means that unlike in the cosinusoidal case, aliasing cannot be avoided here (unless the signal is low-pass filtered before being sampled). See also Remark 9 in the electronic supplementary material, Appendix C.

## Data accessibility

Appendices A–D, as well as the two interactive Mathematica^®^ applications, their user's guide and a glossary of the main terms have been uploaded as the electronic supplementary material. This paper has been significantly shortened in accordance with the reviewers' remarks. Interested readers can find the full original version of this paper, including more detailed figures and examples, in the tutorial [16].
