# Supplementary material for: Sub-Nyquist artefacts and sampling moiré effects
Source: R Soc Open Sci. 2015 Mar 18;2(3):140550. doi: 10.1098/rsos.140550 (PMC4448827; doi:10.1098/rsos.140550)
Supplement: Appendies A-D of the main paper. [file rsos140550supp1.pdf]

Appendices

Appendix A: Moiré theory notations and terminology

In this appendix we provide a brief review of some basic notions and terms from the moiré theory that are being used in the present paper. Interested readers may find further details in publications on the moiré theory such as [6].

The moiré effect is a well-known phenomenon which occurs when two or more repetitive structures (such as periodic signals, waves, line gratings, etc.) are superposed. It consists of a new pattern of alternating dark and bright areas which is clearly observed at the superposition, although it does not appear in any of the original structures (Fig. A1). Usually moiré effects are studied in two-dimensional settings, but the moiré theory is general and it covers also one-dimensional settings (as in the present paper).

Moiré effects may occur in different circumstances. For example, *superposition moirés* occur in the superposition (e.g. overprinting) of two or more periodic structures. *Sampling moirés* occur in the sampling process when an original periodic function $g(x)$ is sampled by a periodic sampling comb. In both cases the interaction between the individual periodic structures gives rise to the resulting moiré effect.

The moiré phenomenon can be explained both in the signal domain and in the spectral domain. From the point of view of the signal domain, the moiré effect occurs due to an interaction between the superposed structures. It results from the geometric distribution of dark and bright areas in the superposition: areas where dark elements of the original structures fall on top of each other appear brighter than areas in which dark elements fall between each other and fill the spaces better (see Fig. A1).

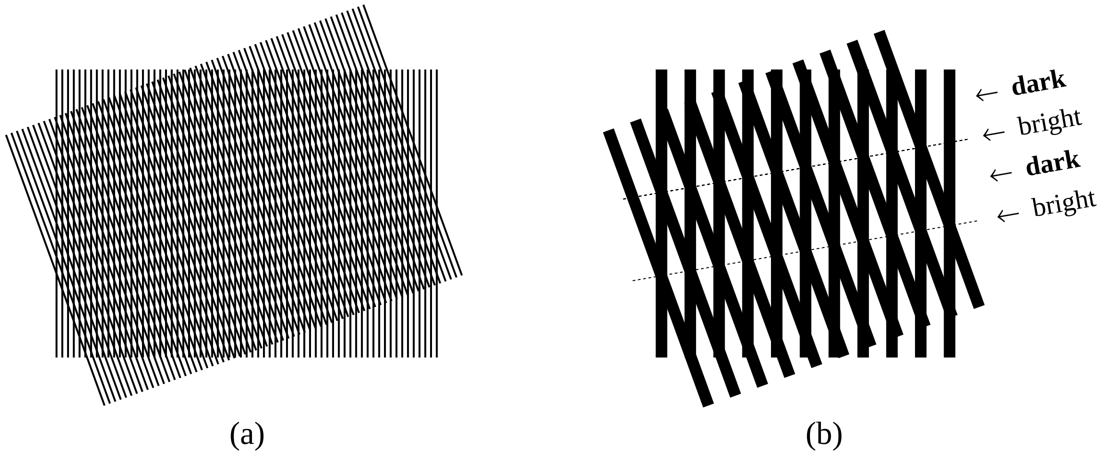

Figure A1: (a) The moiré effect in the superposition of two identical, mutually rotated line gratings. It consists of a new pattern of alternating dark and bright areas that are only formed in the superposed area. (b) Enlarged view.

The spectral or Fourier domain explanation, on its part, is based on the frequencies involved in each of the N superposed layers, in the corresponding frequency spectrum (see Fig. A2). The superposition of layers in the signal domain is viewed mathematically as the *product* of the given structures (where 0 represents black, 1 represents white, and intermediate values represent in-between shades). Therefore, in the Fourier frequency domain, the spectrum of the superposition is the *convolution* of the spectra of the N individual layers. This convolution often gives rise to new low frequencies which did not exist in any of the original layers; these frequencies are the spectral-domain representation of the moiré effects that we see in the superposition, back in the signal domain.

Since our original structures are periodic, their spectra consist of impulses (the base frequency of the structure and its higher harmonics, if any). Considering the impulse locations in the spectrum as vectors emanating from the origin, the impulse locations in the convolution of N such spectra can be viewed as a set of vector sums: Each frequency in the convolution is a vector sum of one vector from the first spectrum, one vector from the second spectrum, ... and one vector from the N-th spectrum: $k_1f_1 + ... + k_Nf_N$, where f_i is the base frequency of the i-th superposed structure and k_if_i is the k_i harmonic (provided that it exists). If any of the new vector sums in the convolution falls close to the spectrum origin, within the visibility circle[1], namely if $k_1f_1 + ... + k_Nf_N \approx 0$, the resulting frequency corresponds to a visible moiré effect in the structure superposition. When the vector sum in question falls exactly on the spectrum origin we get the *singular state* of the moiré, where the moiré period is infinitely long and therefore not visible. But as soon as the vector sum starts moving away from the origin, the corresponding moiré "comes back from infinity" and becomes again visible with a long period. As the moiré vector (the vector sum in question) moves away from the origin to any direction, the frequency of the moiré gradually increases and its period decreases, until finally the moiré effect becomes invisible and disappears.

In the moiré theory, the moiré effect generated between N periodic structures having frequencies f_i, $i = 1,...,N$ when $k_1f_1 + ... + k_Nf_N \approx 0$ is traditionally called the $(k_1,...,k_N)$-moiré effect, since it is generated by an interaction between the k_1-harmonic frequency of the first signal, the k_2-harmonic frequency of the second signal, and so forth (see Sec. 2.8 in [6]). For example, the moiré effect generated between two structures when $f_1 \approx f_2$, so that $f_1 - f_2 \approx 0$, is called a (1,-1)-moiré effect (Fig. A2), and the moiré effect generated between two structures when $f_1 \approx 2f_2$, so that $f_1 - 2f_2 \approx 0$, is called a (1,-2)-moiré effect (Fig. A3).

Note that the situation in the one-dimensional setting is analogous, except that all the frequency vectors in the spectral domain and all the periodicities in the signal domain only take place along the horizontal axis. The two-dimensional Fig. A4 illustrates a one-dimensional setting which has been artificially extended to the two dimensional plane.

---

[1] The *visibility circle* is a circular region with a small radius about the spectrum origin, which includes all frequencies that are small enough to be visible (under some given viewing conditions). In the moiré theory the visibility circle is usually drawn assuming viewing conditions under which only the moiré effects are visible, but all the original frequencies $f_1,...,f_N$ remain beyond the visibility circle. See, for example, [6, Chapter 2].

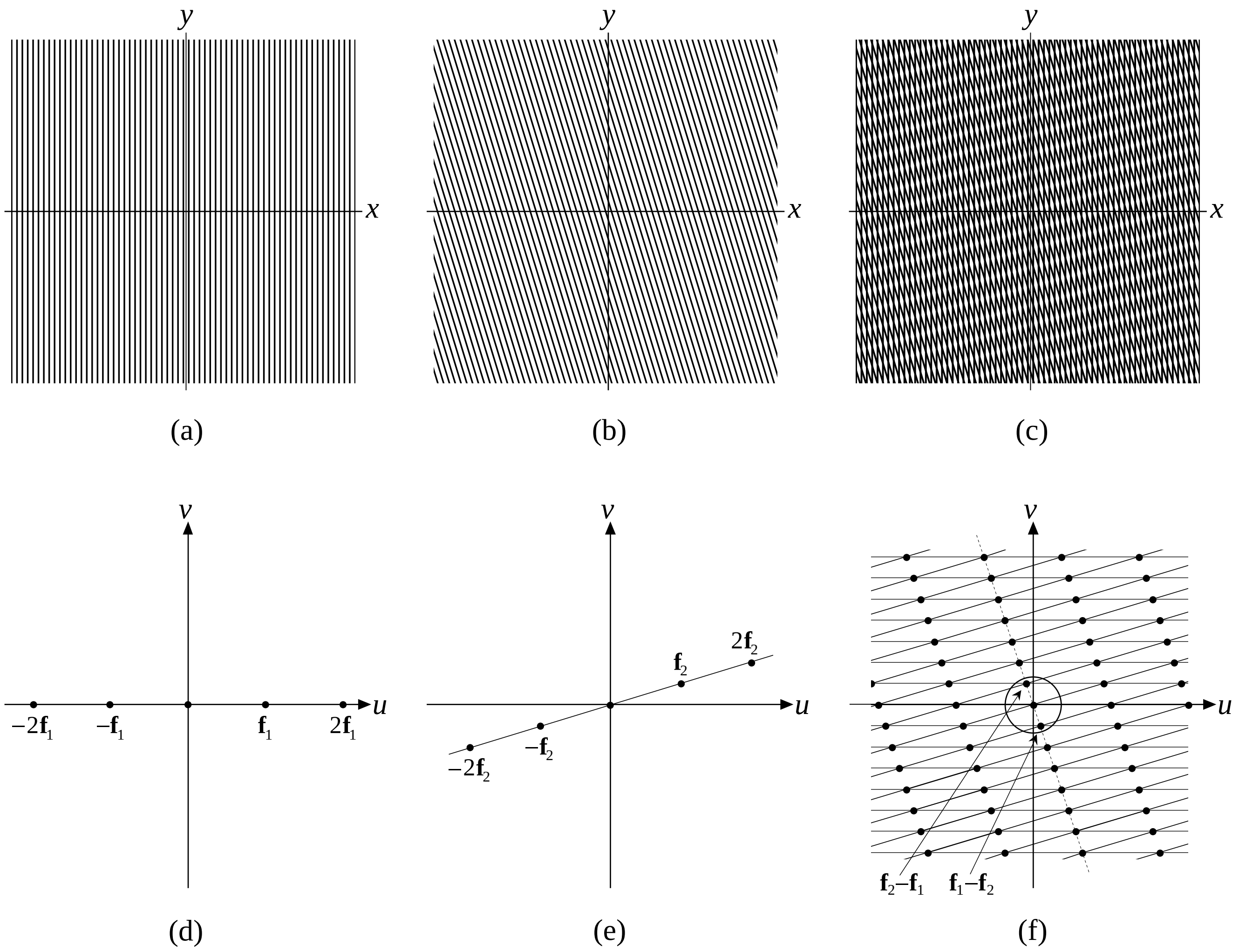

Figure A2: Line gratings (a) and (b) and their superposition (c) in the signal domain; the respective spectra are the infinite combs shown in (d) and (e), and their convolution (f). Note in the center of spectrum (f) the new impulse pair near the origin whose frequency vectors are $\mathbf{f}_1 - \mathbf{f}_2$ and $\mathbf{f}_2 - \mathbf{f}_1$. This is the fundamental impulse pair of the (1,-1)-moiré seen in the superposition (c). Note that the spectra (d)-(f) are purely impulsive; the straight lines connecting between the impulse locations have been added for didactic reasons only, to clarify the geometric structure of these spectra. The dotted line in (f) indicates the infinite impulse comb representing the moiré.

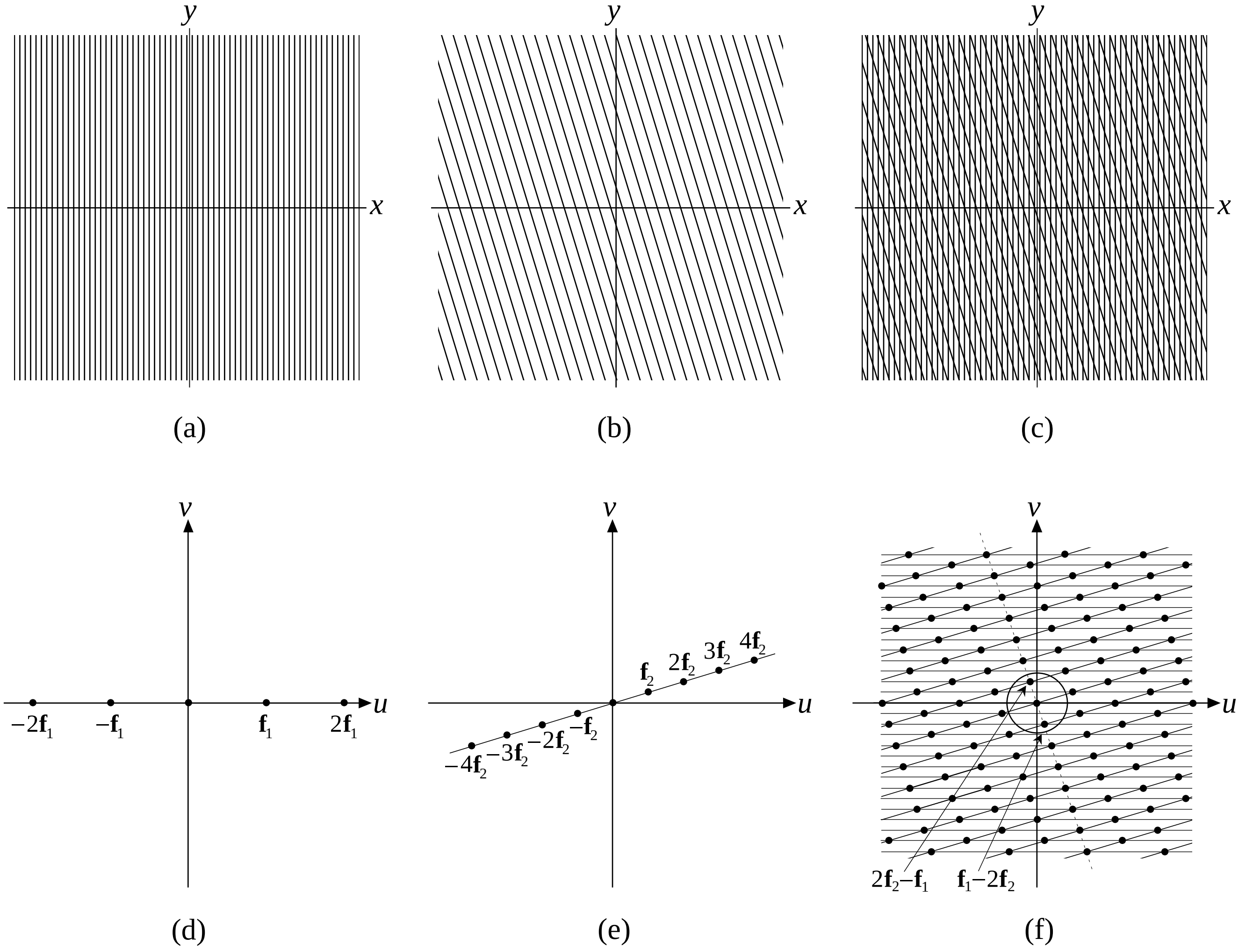

Figure A3: Line gratings (a) and (b) as in Fig. A2 but with (b) having half the frequency, and their superposition (c); the respective spectra are the infinite combs shown in (d) and (e), and their convolution (f). Note in the center of spectrum (f) the new impulse pair near the origin whose frequency vectors are $\mathbf{f}_1 - 2\mathbf{f}_2$ and $2\mathbf{f}_2 - \mathbf{f}_1$, which originate from the second harmonic of $\mathbf{f}_2$. This is the fundamental impulse pair of the (1,-2)-moiré seen in the superposition (c). The dotted line in (f) indicates the infinite impulse comb representing the moiré.

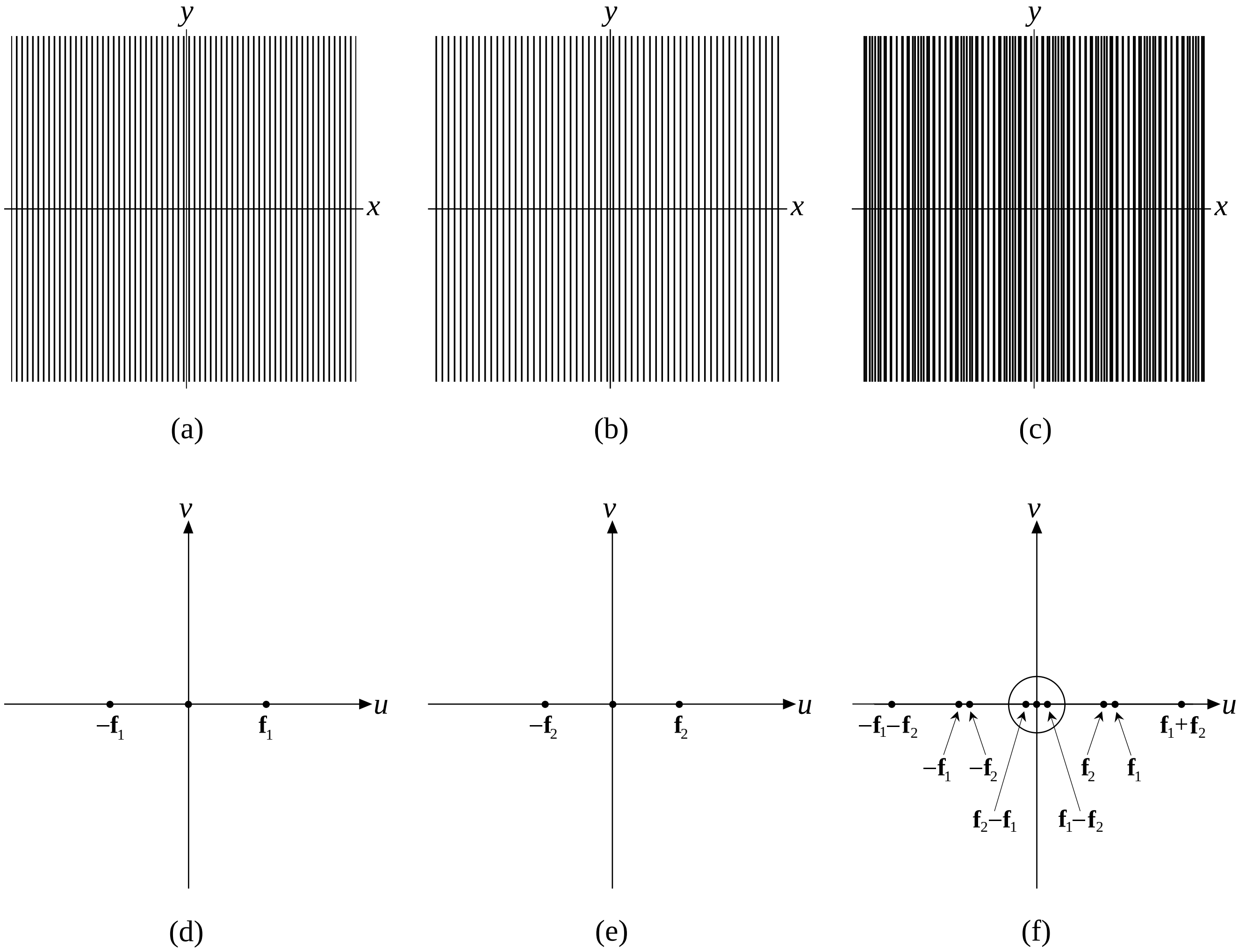

Figure A4: Vertical line gratings (a) and (b) and their superposition (c) in the signal domain; the respective spectra are shown in (d) and (e) and their convolution (f). Since in this case all the frequency vectors in the spectral domain fall on the horizontal *u* axis, only the first-harmonic frequencies are shown, to avoid cluttering along this axis. Note in the center of spectrum (f) the new impulse pair near the origin whose frequency vectors are $\mathbf{f}_1 - \mathbf{f}_2$ and $\mathbf{f}_2 - \mathbf{f}_1$. This is the fundamental impulse pair of the (1,-1)-moiré seen in the superposition (c). The present figure illustrates a one-dimensional setting, that has been extended to the two dimensional plane for an improved visibility. The true one-dimensional version of this figure consists of the cross sections passing through the horizontal axes of (a)-(f).

The true one-dimensional setting consists of a cross section through the horizontal axis in both signal and spectral domains. (Note however that this is not true for figures such as A1-A3, which indeed contain true two-dimensional information).

Now, in the case of *sampling moirés* (moiré effects that are generated when sampling a periodic signal $g(x)$ of frequency f using a sampling frequency of f_s) only N=2 structures are involved, $g(x)$ and the sampling comb. Therefore, using the traditional moiré-theory notation, when $mf_s + nf \approx 0$ we obtain a (m,n)-sampling-moiré effect. This moiré is generated in the sampled signal $g(x_k)$ due to an interaction between the m-th harmonic of the sampling frequency f_s and the n-th harmonic of f, the fundamental frequency of $g(x)$.[2] See, for example, the (1,-1)-sampling moiré in Figs. 2 and 5, where $f_s - f \approx 0$. (Note that in these figures the frequencies f and f_s fall outside the spectrum range, and only the folded over moiré frequencies are visible, near the origin).

Sampling-theory considerations can be often interpreted in a dual way using moiré theory considerations, and vice versa. This duality sheds a new light on the results in question thanks to the cross-fertilisation between these two points of view.

---

[2] Of course, this interaction may only occur if the spectrum (Fourier decomposition) of the signal $g(x)$ contains the n-th harmonic of the signal's frequency f. Otherwise, no sampling moiré may be generated here (although a (m/n)-order sub-Nyquist artifact *may* occur, since $mf_s + nf \approx 0$ is equivalent to $f \approx \frac{m}{n} f_s$).

Appendix B: The phase terminology for periodic functions

Let us introduce some notations and terms in connection with the phase of periodic functions.

Suppose we are given a cosine function $g(x) = \cos(2\pi fx)$ with frequency f and period $p = 1/f$, and that we shift it by a along the x axis:

$$g(x+a) = \cos(2\pi\tfrac{1}{p}(x+a)) = \cos(2\pi\tfrac{1}{p}x + 2\pi\tfrac{1}{p}a) \qquad \text{(B.1)}$$

We define $\varphi = 2\pi\frac{1}{p}a = 2\pi fa$ as the *phase* of our shifted cosine. We therefore have:

$$= \cos(2\pi\tfrac{1}{p}x + \varphi) \qquad \text{(B.2)}$$

As we vary the shift a from 0 to p, the phase φ of our cosine varies from 0 to 2π, and completes one full cycle of 2π. Note that with respect to φ our cosine is cyclical modulo 2π: for any integer k, $\cos(2\pi\frac{1}{p}x + [\varphi + 2\pi k]) = \cos(2\pi\frac{1}{p}x + \varphi)$. Hence, a phase of $2\pi k$ is equivalent to the phase 0.

We now define the *relative shift* (or the *period shift*) $\phi = \frac{a}{p} = fa$ as the shift undergone by $g(x+a)$, expressed as a number (integer or not) of periods p of $g(x)$. We therefore have from Eq. (B.1):

$$= \cos(2\pi\tfrac{1}{p}x + 2\pi\phi) \qquad \text{(B.3)}$$

Note that with respect to the relative shift ϕ our shifted cosine is cyclical modulo 1: for any integer k, $\cos(2\pi\frac{1}{p}x + 2\pi[\phi + k]) = \cos(2\pi\frac{1}{p}x + 2\pi\phi)$. As we vary the shift a from 0 to p, the relative shift ϕ varies from 0 to 1, completing one full cycle of the cosine. Hence, a relative shift of $\phi = k$ is equivalent to $\phi = 0$.

Because we have $\varphi = 2\pi\phi$ it is clear that φ and ϕ are equivalent, and both of them may be used to denote the phase of any shifted periodic function $g(x+a)$. However, the phase φ is mainly useful in functions such as sine or cosine, where the constant 2π has indeed an intrinsic significance. In other periodic functions such as a square wave, a triangular wave, etc., where 2π does not explicitly appear in the function definition itself, the use of ϕ to designate the phase may be more natural.

In the case of a general periodic function $g(x)$ having the Fourier series expansion

$$g(x) = \textstyle\sum_{l=-\infty}^{\infty} c_l\, e^{i2\pi lfx} \qquad \text{(B.4)}$$

the influence of a shift of a on the phase of $g(x)$ is expressed in terms of the relative shift ϕ harmonic by harmonic, as follows:

$$g(x+a) = \textstyle\sum_{l=-\infty}^{\infty} c_l\, e^{i2\pi lf(x+a)} = \sum_{l=-\infty}^{\infty} c_l\, e^{i2\pi lfx + i2\pi lfa}$$

$$= \textstyle\sum_{l=-\infty}^{\infty} c_l\, e^{i2\pi lfx + i2\pi l\phi} \qquad \text{(B.5)}$$

Note that here, too, with respect to the relative shift ϕ our shifted function (B.5) is cyclical modulo 1: for any integer k,

$$\sum_{l=-\infty}^{\infty} c_l\, e^{i2\pi lfx\, +\, i2\pi l[\phi+k]} = \sum_{l=-\infty}^{\infty} c_l\, e^{i2\pi lfx\, +\, i2\pi l\phi\, +\, i2\pi lk}$$

$$= \sum_{l=-\infty}^{\infty} c_l\, e^{i2\pi lfx\, +\, i2\pi l\phi}\ e^{i2\pi lk} = \sum_{l=-\infty}^{\infty} c_l\, e^{i2\pi lfx\, +\, i2\pi l\phi}$$

since $e^{i2\pi lk} = \cos(2\pi lk) + i\, \sin(2\pi lk) = 1$ for any integers k and l [9, p. 96]. As we vary the shift a from 0 to p, the relative shift ϕ varies from 0 to 1, completing one full cycle. This result will be used in Appendix D in the supplementary material.

More detailed information on the phase terminology for periodic functions can be found in [6, Secs. 7.3 and C.4]. Interested readers may also have their own hands-on experience using the provided interactive applications, which allow one to gradually vary ϕ between 0 and 1 and observe the resulting effects both in the signal and in the spectral domains.

Appendix C: Miscellaneous remarks

In this appendix we provide some further remarks that consolidate the results obtained so far, and shed new light on various aspects of the sub-Nyquist artifacts.

Remark 1 (*even and odd ripples*):

When the value of n is odd, like in the (1/3)-order sub-Nyquist artifact, we get an odd number n of interlaced envelopes (see Fig. 4). Consequently, the modulation effect we obtain here is not vertically symmetric as in Fig. 3 (its maxima and minima, i.e. its top ripples and bottom ripples, are not synchronized but rather intermittent). We will henceforth call the synchronized type of ripples "even ripples", and the non-synchronized type "odd ripples". ■

Remark 2 (*the beating effect due to the modulating envelopes*):

Suppose we are sampling a cosinusoidal signal and that we plot the results, much like on the display of an oscilloscope, by connecting consecutive samples with straight line segments. When plotting the sampled signal densely, i.e. when the number of samples per envelope-period p_{env} is relatively high, a sub-Nyquist artifact may confer to the sampled signal a typical wavy or fringy appearance. This beating or ripple effect is best observed when we plot the sampled signal $g(x_k)$ alone, without the original continuous-world cosine function $g(x)$ (note that in Figs. 1-6 we always overprinted the original continuous signal, too, for didactic reasons). This beating effect is illustrated for the cases of (m/n) = (1/1) and (1/2) in Figs. C1 and C2, respectively. In each of these figures the left-hand column shows the sampled version of the continuous cosine function $g(x) = \cos(2\pi fx)$, and the right-hand column shows the sampled version of the continuous square-wave function $g(x) = \text{wave}(fx)$ having the same frequency f. In all cases the sampling frequency remains $f_s = 8$, and the only difference between consecutive rows is in the frequency f of the original continuous function, as indicated in each row. Figs. C1-C2 are therefore similar to our previous Figs. 2-6, but they are plotted 8 times more densely and without the original continuous-world signal itself, which was omitted in order not to obscure the sampled signal. Because the sampled signal is plotted here more densely than in Figs. 2-6, the beating or ripple effect due to the modulating envelopes is more clearly visible, even without highlighting the envelope curves themselves as we did in Figs. 2-6.[3] In the cosinusoidal case, the depth of this ripple effect (i.e. the amplitude of the resulting corrugations along the top or bottom boundary of the sampled signal) depends on the values of n and m: As we can see in the left-hand column of Fig. C2, when $m = 1$ and $n = 2$, i.e. in the case of the simple (1/2)-order sub-Nyquist artifact, this depth equals half of the amplitude of the original continuous cosinusoidal signal (the vertical distance between the node and the maximum of the two modulating envelopes). But the depth of the ripple effect decreases as the number n increases. This depth can be readily found by calculating the height of the intersection points between the interlaced envelopes, and subtracting it from the maximum height of the envelopes. ■

[3] Note that the dark geometric patterns that may appear when plotting the sampled signal very densely are only display artifacts. They may differ depending on the display devices being used, and they disappear when plotting the same figure at a larger scale.

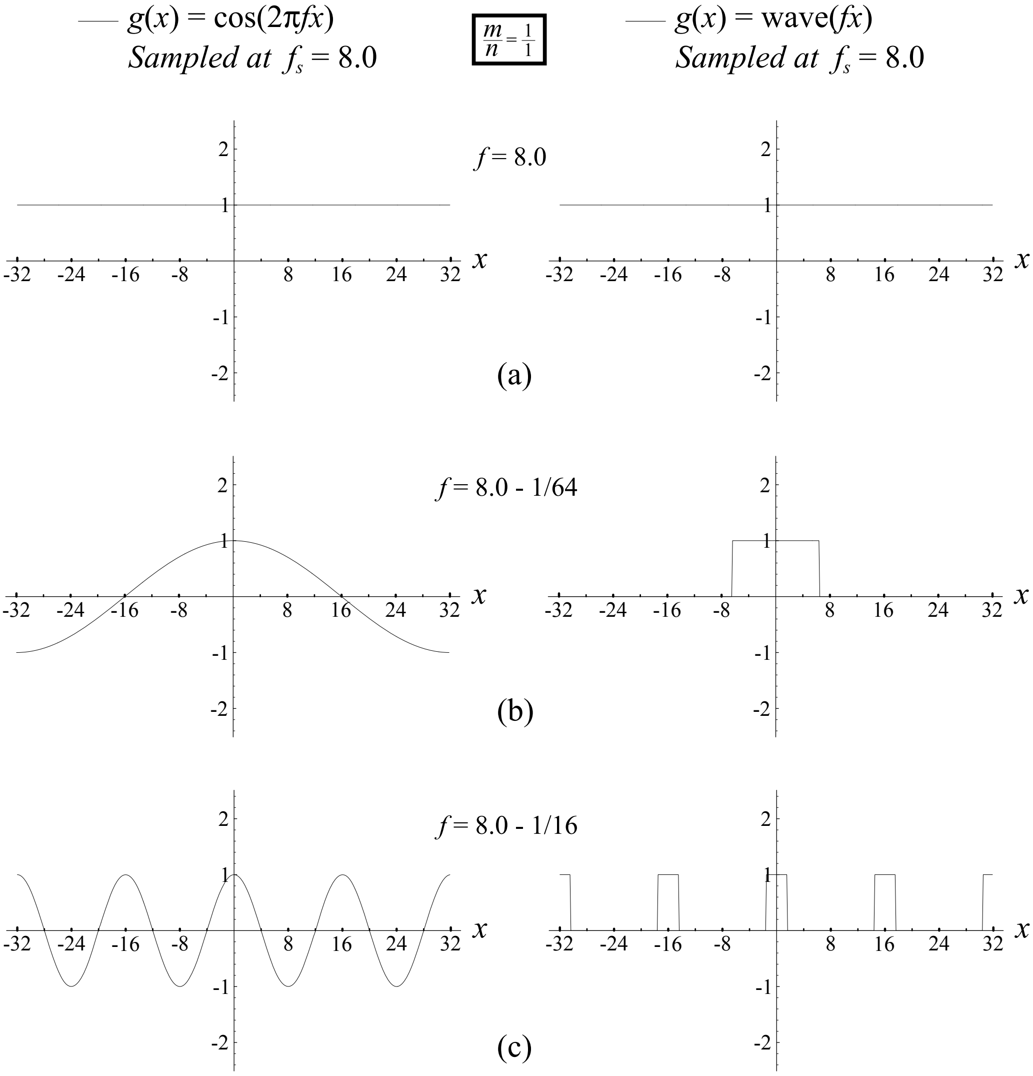

Figure C1: The (1/1)-order artifact (which is, in fact, a true first-order sampling moiré) as it appears in densely plotted periodic signals. Left-hand column: the periodic signal $g(x) = \cos(2\pi fx)$ having frequency f, after being sampled with a sampling frequency of $f_s = 8$ (i.e. with a sampling interval of $\Delta x = 1/f_s = 1/8$). Right-hand column: the periodic square wave signal $g(x) = \text{wave}(fx)$ (with opening ratio of $\tau/p = 1/5$) having the same frequency f, after being sampled with the same sampling frequency of $f_s = 8$ (and sampling interval of 1/8). Each row in the figure shows both of the sampled signals for the following values of f (frequency of the signal $g(x)$): (a) $f = f_s$ (the singular state). (b) $f = f_s - 1/64$. (c) $f = f_s - 1/16$. All sampled signals are plotted here as *connected line plots*, meaning that consecutive sample points $g(x_k)$ are connected by straight line segments (see [7, Sec. 1.5.1]), just as on the display of an oscilloscope. The signals are plotted much more densely than in Figs. 2 and 5 (note that we use here the same sampling interval $\Delta x = 1/8$ as in those figures but an 8-fold larger sampling range, and hence 8 times more samples). Unlike in Figs. 2-6 the original continuous signal is not shown here, in order not to obscure the sampled signal itself.

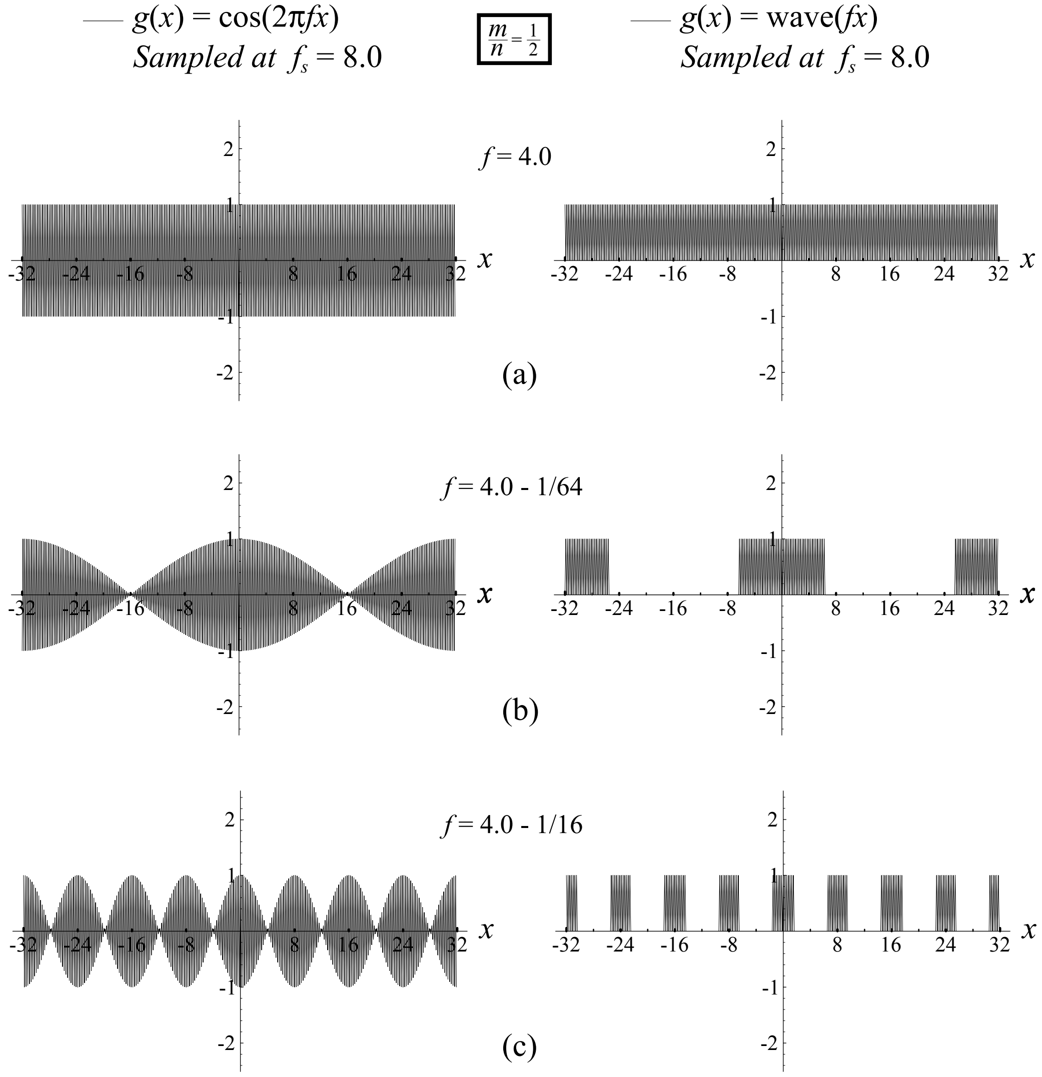

Figure C2: The (1/2)-order sub-Nyquist artifact as it appears in densely plotted periodic signals. This figure is similar to Fig. C1, except for the signal-frequency f being used in each row: (a) $f = \frac{1}{2}f_S$ (the singular state). (b) $f = \frac{1}{2}f_S - 1/64$. (c) $f = \frac{1}{2}f_S - 1/16$. The highly visible (1/2)-order sub-Nyquist artifact is generated because consecutive points of each sampled signal alternately jump from one of the n = 2 modulating envelopes to the other, as shown in greater detail in Figs. 3 and 6, respectively.

Remark 3 (*significance of the sign of* ε):

Now that we know how to treat sub-Nyquist artifacts in any periodic function, including functions having an asymmetric period, we are ready to see the effect of the sign of ε. In all our figures so far we always used negative values of ε, meaning that the frequency f of our given periodic function was varying below its singular value, $(m/n)f_s$.[4] Because our given function (cosine or square wave) was symmetric, the results obtained when using positive values of ε were exactly the same. Let us see now what happens when our given function $g(x)$ is asymmetric. Consider, for example, the continuous periodic sawtooth function $\text{saw}(x) = x \bmod 1$, which consists of a sequence of asymmetric "teeth" having period 1 (or its normalized version, $g(x) = \text{saw}(fx)$, whose frequency is f). Fig. C3 shows in its left-hand column what happens to the sampled signal $g(x_k)$ when we take positive values of ε, and in its right-hand column what happens when we take negative values of ε (see also Fig. C4, which shows the spectral domain, too). Although the behaviour in both cases remains globally the same, the orientation of the sub-Nyquist artifact is flipped (mirror-imaged) when ε is negative (i.e. when f is slightly below the singular value $(m/n)f_s$). In other words, as f sweeps *backward* along the frequency axis and approaches the singular state from above, the sub-Nyquist artifact gradually becomes bigger, until it becomes infinitely big and disappears when f precisely reaches the singular value $(m/n)f_s$. Then, when f pursues its way backwards below the singular value, the (m/n)-order sub-Nyquist artifact "comes back from infinity" and becomes again visible, but this time with an inversed orientation. This behaviour is well known in the moiré theory (see, for example, Fig. C.24 in [6]), and it is interesting to see that it is preserved in the case of sub-Nyquist artifacts, too. ■

This result also means that the magnification rate f/ε mentioned following Theorem 6.1 is in fact sign-sensitive, where a negative sign simply means magnification with mirror-inversion.

Remark 4 (*omnipresence of modulating envelopes*):

It is important to note that modulating envelopes can be traced through the samples of our continuous-world signal in all circumstances, whether the signal's frequency f is close to $(m/n)f_s$ or not (note that Theorems 5.1 and 6.1 hold for *any* value of ε, be it small or large). Moreover, for any given f and f_s, different sets of modulating envelopes belonging to different m, n values can be always traced through the very same samples $g(x_k)$, $k = 0, 1, 2,...$[5] Nevertheless, these modulating envelopes only become relevant when f is sufficiently close to $(m/n)f_s$, i.e. when $\varepsilon = f - (m/n)f_s$ is small, thanks to the visible beating or ripple effect that is generated by the (m/n)-order sub-Nyquist artifact in these cases. Furthermore, since rational numbers m/n can be always found that closely approximate any real number r, be it rational or irrational, it turns out that (m/n)-order modulation effects may occur at any frequency f along the frequency axis. But of course, the larger the integer number n (and hence the number of interlaced envelopes),

---

[4] The reason we have been using negative values of ε is that in Eq. (4.4), i.e. in the case of the (1/2)-order sub-Nyquist artifact, we wanted f to remain below the Nyquist limit of $\frac{1}{2}f_s$. We then continued using negative values of ε in all the other cases, too, for the sake of consistency.

[5] Note that for any given f and f_s there exist infinitely many values of m,n and ε that satisfy Eq. (5.5), $f = (m/n) f_s + \varepsilon$: For any chosen m and n, there exists a corresponding ε value that satisfies the equation.

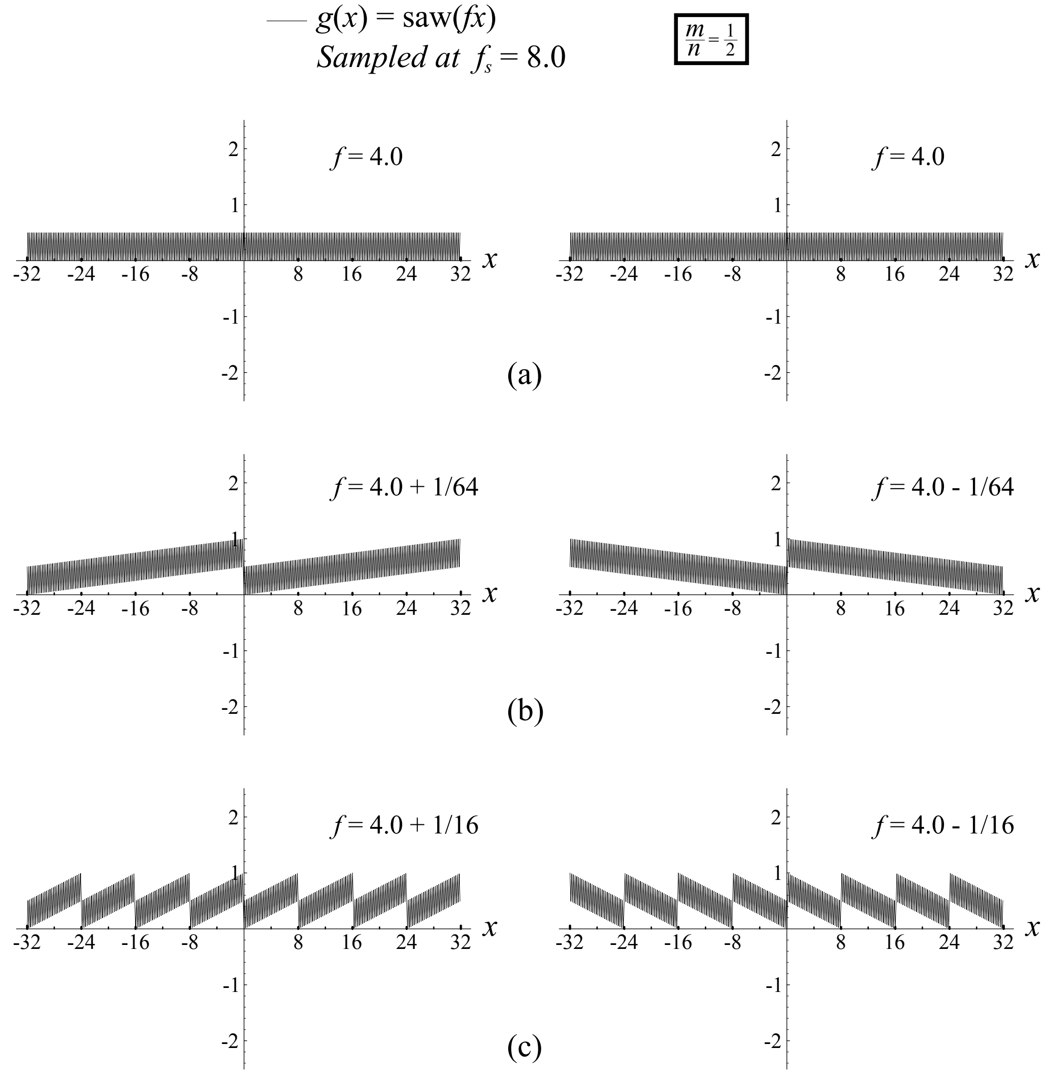

Figure C3: The (1/2)-order sub-Nyquist artifact as it appears in densely plotted periodic sawtooth signals. This figure illustrates the role of the sign of ε: It shows in the right-hand column the periodic sawtooth signal $g(x)$ = saw(fx) = fx mod 1 (where f is the signal's frequency) after being sampled with a sampling frequency of f_s = 8 (i.e. with a sampling interval of Δx = 1/8), with the following values of f: (a) $f = f_s$ (the singular state). (b) $f = f_s$ – 1/64. (c) $f = f_s$ – 1/16. The left-hand side column shows what happens in the very same cases if we only change the sign of ε so that the values of f become: (a) $f = \frac{1}{2}f_s$ (the singular state). (b) $f = \frac{1}{2}f_s$ + 1/64. (c) $f = \frac{1}{2}f_s$ + 1/16. Note that the modulating envelopes in both columns are simply stretched (and shifted) versions of $g(x)$, but in the right-hand column (where the sign of ε is negative) they are also mirror-inversed with respect to the original sawtooth $g(x)$. Here, too, the highly visible (1/2)-order sub-Nyquist artifacts are generated because consecutive points of each sampled signal alternately jump from one of the n = 2 modulating envelopes to the other. See also Fig. C4, in greater detail.

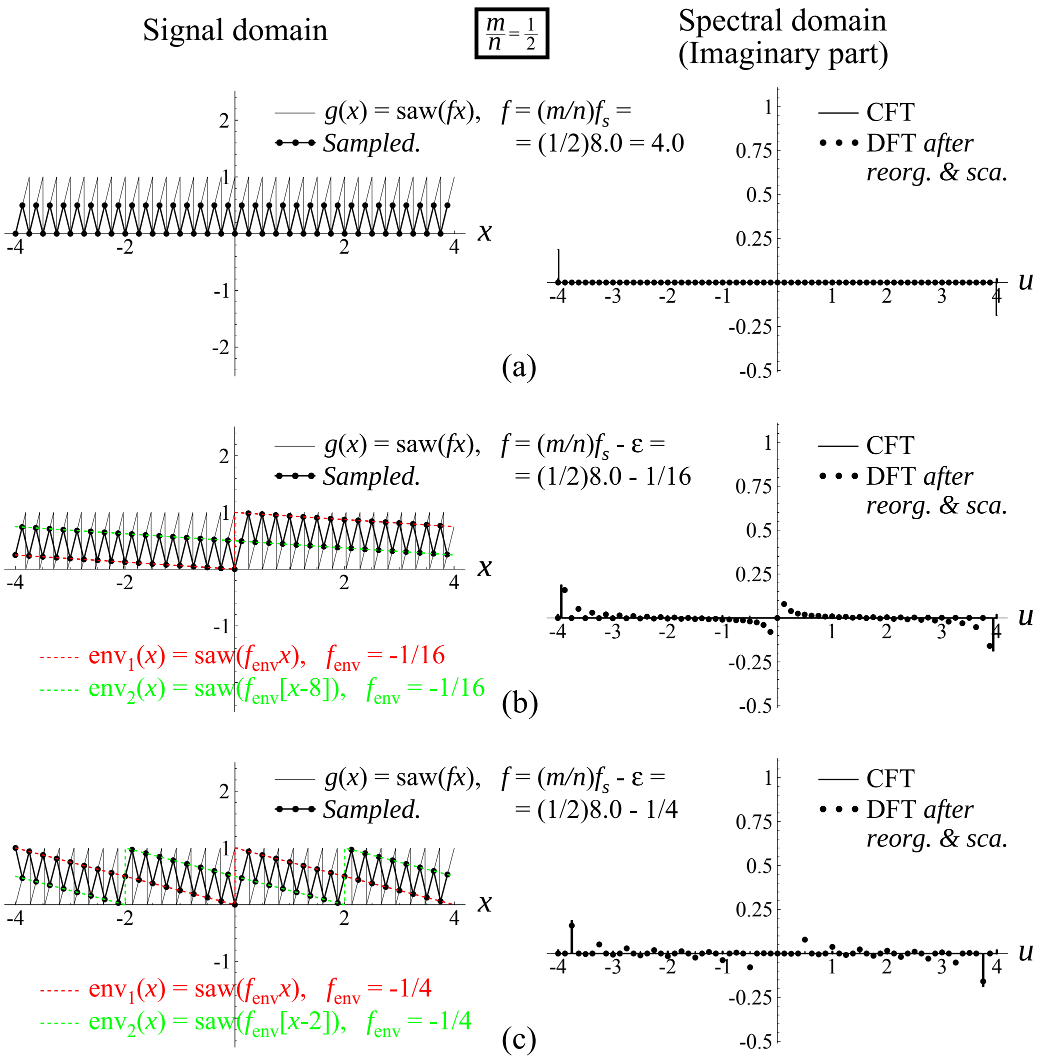

Figure C4: A more detailed view of the (1/2)-order sub-Nyquist artifact that is shown in the right-hand column of Fig. C3, along with the corresponding spectral domain representations. Note that saw(fx) can be seen as an odd function plus the constant 0.5; the spectrum of an odd real-valued function is odd and imaginary-valued [17, p. 15], and the spectrum of the constant 0.5 is a real-valued impulse of strength 0.5 at the origin (the real-valued part of the spectrum is not shown here). Compare with the cosine and square-wave counterparts in Figs. 3 and 6, respectively; note that the envelope frequency f_{env} has negative ε values in all of these figures, but it only matters in the present figure, where $g(x)$ is not symmetric.

the less visible and prominent the envelopes become. The reason is that when several envelopes are intermingled together it becomes more difficult for the eye to detect and follow each of the envelopes separately, i.e. to detect a visible order within the sampled points. Furthermore, in the case of a sampled cosinusoidal signal, in larger values of n the depth of the ripple effect becomes smaller (see Remark 2 above) and hence the beats are less conspicuous. Thus, although modulating envelopes are always present in the sampled signal $g(x_k)$, in practice the resulting beating effect (sub-Nyquist artifact) is only visible for relatively low values of n. In other words, the sampled points of a periodic function $g(x)$ are always located on (i.e. they can always be decomposed into) various sets of interlaced envelopes; but these envelopes truly become visible only when the value of n is relatively low.

We therefore obtain the following result: If we slowly vary the frequency f of the original periodic function $g(x)$ and let it sweep along the frequency axis, we will only find a limited number of zones in which f is close to a $(m/n)f_s$ value (singular point) with a small n, where the envelopes give a clearly visible beating effect. Although infinitely many singular points $(m/n)f_s$ exist throughout the frequency axis (in fact, they are everywhere dense along this axis), only few of them will give a "dangerous zone" that f should avoid when sweeping along the frequency axis in order not to generate visible sub-Nyquist artifacts.

The dual result obtained by considering our question the other way around may be even more interesting: If we fix the frequency f of the original periodic function $g(x)$ and let the sampling frequency f_s slowly vary and sweep along the frequency axis, we will only find a limited number of "dangerous zones" of sampling frequencies f_s that should be avoided when sampling our given periodic function $g(x)$. These "dangerous zones" occur when f_s is located around one of the frequencies $(n/m)f$ with small integers m, n (note that $f \approx (m/n)f_s$ is equivalent to $f_s \approx (n/m)f$). ■

Remark 5 (*singular state of the (m/n)-order sub-Nyquist artifact*):

When the frequency f of the original continuous-world function $g(x)$ equals exactly $(m/n)f_s$, i.e. when $\varepsilon = 0$, the period of the (m/n)-order sub-Nyquist artifact becomes infinitely large, and hence the corresponding beating or ripple is no longer visible and the sampled signal becomes uniform and periodic, as shown in rows (a) of Figs. 2 and on. We call this critical frequency the *singular state* of the (m/n)-order sub-Nyquist artifact, in analogy to the singular state of the (m,n)-moiré effect (see Appendix A, or Sec. 2.9 in [6]). And indeed, just like in the case of the moiré, as soon as the frequency f starts moving away from the singular state (to either direction), the (m/n)-order sub-Nyquist artifact "comes back from infinity" and becomes again visible, with a long-period ripple. As f moves further away from the singular state, i.e. as $|\varepsilon|$ gradually increases (see rows (b)-(c) in Figs. 2 and on), the period of the beating or ripple effect becomes smaller, until finally it becomes very small and completely disappears.

As the frequency f sweeps along the frequency axis, it passes through the singular states of many different (m/n)-order sub-Nyquist artifacts. Each time, a similar scenario occurs as the frequency f approaches the singular state, coincides with it, and then passes beyond it. Interested readers may get a vivid, dynamic demonstration using the provided

interactive applications, which allow one to slowly vary the frequency f and observe the resulting effects on the sampled signal. It should be noted, however, that the sub-Nyquist artifacts we thus meet on our way while varying f are all different, depending on their m,n values: They differ in their prominence, their number of modulating envelopes, their even or odd nature (see Remark 1), etc.

Because the singular state of an (m/n)-order sub-Nyquist artifact occurs at the frequency $f = (m/n)f_s$, it follows that for any frequency f of the original function $g(x)$, close-by (m/n)-order singular states will always exist in the neighbourhood of f for some integer pairs m, n that satisfy $f \approx (m/n)f_s$, i.e. $m/n \approx f/f_s$. However, as explained in Remarks 2 and 4, most of the resulting (m/n)-order sub-Nyquist artifacts are negligible and hardly visible, since their n value is relatively high. ■

Remark 6 (*how to identify to which (m/n)-order belongs a given case*):

Suppose we are given a beating or ripple effect that occurs when sampling a periodic continuous-world function $g(x)$ of frequency f using the sampling frequency f_s. How can we find out to which (m/n)-order sub-Nyquist artifact belongs our case? An easy, empirical way consists of the following steps: We first plot the given case (possibly after scaling the values of f and f_s to adapt them to our plotting conventions, but obviously using the same scaling factor for both frequencies so as to keep the ratio f/f_s unchanged; this frequency scaling corresponds, in fact, to a horizontal stretching of the plot along the x axis). Then, we slightly increment or decrement f by a very small value δ, and plot the resulting sampled signals having the frequencies $f + \delta$ and $f - \delta$. The aim of this step is to determine to which direction we must vary f along the frequency axis in order to increase the beat's period (i.e. in order to get closer to the singular frequency of our given beating effect). We then continue varying f very slowly in that direction, each time re-plotting the sampled signal, and we stop when the beats become infinitely large and hence invisible (note that beyond this critical value of f the beats start to reappear once again). Having thus identified the singular frequency f belonging to our case, we simply have to find which mutually prime integers m and n satisfy $m/n = f/f_s$. Note that different (m/n)-order cases may possess very close singular frequencies, so the step δ we are using must be very small in order not to miss the correct singular state; often several digits beyond the decimal point may be required.

As an illustrative example, let us apply this method to analyze the nature of the beating effect reported in [4], where $f_s = 12170$ and $f = 1367$. We first rescale these values to adapt them to our plotting conventions, in which we are using $f_s = 8$; this is done by taking $f = 1367 \times 8/12170 = 0.8986$. Now, by slowly varying f and each time plotting the resulting sampled signal, as explained above, we find that the corresponding singular state occurs at $f = 0.888...$ Therefore we obtain $m/n = f/f_s = 0.111...$, and we conclude that $(m/n) = (1/9)$. This is, indeed, an odd-type ripple (see Remark 1), having 9 interlaced modulating envelopes. Note, however, that the case shown in Fig. 7 of [4] is different, and corresponds to an odd-type (1/5)-order sub-Nyquist artifact having 5 interlaced envelopes, as we can clearly see in that figure. ■

Remark 7 (*coexistence of sub-Nyquist artifacts with leakage*):

Although the sub-Nyquist artifact is a discrete-world phenomenon, it is not affected by leakage (the leakage phenomenon is explained, for example, in [7, Ch. 6] or in [9, pp. 98-107]). In other words, the modulating envelopes in the signal domain remain perfectly accurate, without any flaws, even in cases where the frequency f and/or the frequency $(m/n)f_s$ do not fall exactly on a discrete point of the DFT spectrum, but rather between two such points (see, for example, Fig. 4). In all of these cases the modulating envelopes in the image domain behave exactly as predicted, and they are not flawed by the existence of leakage. ■

Remark 8 (*coexistence of sub-Nyquist artifacts with aliasing*):

Similarly, sub-Nyquist artifacts may also coexist with aliasing (folding-over). In other words, sub-Nyquist artifacts occur even when the frequency f of the original function $g(x)$ (or its higher harmonics, if any) fall beyond half of the sampling frequency, i.e. beyond the boundaries of the DFT spectrum (note that the DFT spectrum always extends between minus and plus half of the sampling frequency [7, p. 73]). And indeed, the modulating envelopes we obtain thanks to the sub-Nyquist artifact remain identical for folded-over or non folded-over frequencies. This is illustrated, for example, in rows (f) and (h) of Fig. 8.13 in [7], which look identical although row (h) is a folded-over counterpart of row (f).[6] Note that when $m > n/2$ so that the frequency $f \approx (m/n)f_s$ is higher than $0.5f_s$, which also includes all cases with $n = 1$ (true moiré effects), aliasing does occur, and the term "sub-Nyquist artifacts" is no longer adequate; but we will continue using it here, too, for consistency reasons and for want of a better term. It should be noted that when a folded-over frequency due to aliasing falls close to the spectrum origin, a true moiré effect becomes visible in the sampled signal (Fig. 1(c)). This occurs, for example, in the (1/1)-order or (2/1)-order cases. ■

Remark 9 (*higher harmonics*):

We have seen that when $n = 1$, the sampled points $g(x_k)$, $k = 0, 1, 2,...$ fall on a single low-frequency curve, meaning that the $(m/1)$-order sub-Nyquist artifact is, indeed, a true $(m,-1)$-moiré effect. We know from the moiré theory (see Appendix A) that true moiré effects may also occur for higher values of n, whenever $mf_s + nf \approx 0$. However, this is only possible if the periodic function $g(x)$ contains the n-th harmonic of its frequency f. For example, a (1/2)-order sub-Nyquist artifact cannot be a true moiré effect when the function being sampled is $g(x) = \cos(2\pi fx)$, since the cosine function does not possess a second harmonic of f. But when the function being sampled is the square wave $g(x) = \text{wave}(fx)$ having frequency f (see Fig. 6), which does have the second harmonic of f, the (1/2)-order sub-Nyquist artifact can be considered as a true (1,-2)-moiré. And indeed, looking at the spectral domain in Fig. 6, we see that the spectra (DFT) of the sampled signals do contain new low frequencies near the origin, that did not exist in the spectra (CFT) of the original continuous signals. These new low frequencies correspond, indeed, to the true (1,-2)-moiré effect. But as expected, this does not occur in the

[6] The (1/2)-sub-Nyquist artifact is indeed unique in that its singular point $(1/2)f_s$ exactly coincides with the Nyquist frequency $0.5f_s$, so that for $\varepsilon < 0$ no aliasing exists (see Figs. 8.13(e),(f) in [7]), while for $\varepsilon > 0$ aliasing does occur (Fig. 8.13(h) in [7]).

cosinusoidal counterpart of Fig. 6, shown in Fig. 3, which remains a pure (1/2)-order sub-Nyquist artifact.

Interestingly, although the (1/2)-order sub-Nyquist artifact of the square wave (see Fig. 6) can be considered as a true moiré effect, the sampled points $g(x_k)$, $k = 0, 1, 2,...$ in its signal domain do jump alternately between two modulating envelopes. And indeed, the mere fact that $g(x_k)$ alternately jumps between several modulating envelopes does not yet mean that the artifact in question is not a moiré effect. In fact, we can say that our example is a *hybrid* case, sharing properties both of a moiré effect (existence of a true low frequency) and of a sub-Nyquist artifact (jumping alternately between several modulation envelopes). Comparing the sampled signals in Figs. 3 and 6 we can find the clue: In Fig. 3, the moving average of the jumpy sampled signal is identically zero, meaning that no low-frequency content is present in the sampled signal. But in Fig. 6, the moving average of the jumpy sampled signal *does* have a true low-frequency content, which precisely corresponds to a true moiré effect. ■

Remark 10 (*on the notations used for moirés and sub-Nyquist artifacts*):

As we have seen throughout this work, sampling moirés and sub-Nyquist artifacts are particular cases of the same phenomenon, and they obey the same mathematical rules. So why don't we adopt the same notations for both cases?

In the moiré theory, the moiré effect generated between N periodic structures having frequencies f_i, $i = 1,...,N$ when $k_1 f_1 + ... + k_N f_N \approx 0$ is traditionally called the $(k_1,...,k_N)$-moiré effect (see Appendix A, or Sec. 2.8 in [6]). For example, the moiré effect generated between two structures when $f_1 \approx f_2$, so that $f_1 - f_2 \approx 0$, is called the (1,-1)-moiré effect. Now, in the case of sampling moirés (moiré effects that are generated when sampling a periodic function $g(x)$ of frequency f using a sampling frequency of f_s) only two structures are involved, $g(x)$ and the sampling comb. Using the traditional moiré-theory notation, when $mf_s + nf \approx 0$ we obtain a (m,n)-moiré effect. But unlike in cases with $N > 2$, when $N = 2$ it is the ratio m/n that plays the main role, and the integer vector notation can be reduced into the (m/n)-notation. And indeed, this notation is particularly convenient and advantageous in the case of sub-Nyquist artifacts (note, for example, the explicit use of the ratio m/n in Theorems 5.1 and 6.1). Nevertheless, when specifically referring to a moiré effect, we prefer to continue using the classical moiré notations, for reasons of consistency with the existing moiré literature. ■

Appendix D: Derivation of Theorem 6.1

Let $g(x)$ be a periodic function with frequency f and period $p = 1/f$. The Fourier series expansion of $g(x)$ is given, using exponential notation, by:

$$g(x) = \sum_{l=-\infty}^{\infty} c_l \, e^{i2\pi lfx} \qquad \text{(D.1)}$$

where the l-th Fourier series coefficient c_l is

$$c_l = (1/p) \int_p g(x) \, e^{-i2\pi lfx} \, dx$$

(see, for example, [6, Sec. A.1] or [18, pp. 4-6, 173-190]).

When we sample $g(x)$ at the sampling frequency f_s, i.e. using a sampling step of $\Delta x = 1/f_s$, we obtain the sampled signal:

$$g(x_k) = \sum_{l=-\infty}^{\infty} c_l \, e^{i2\pi lfx_k} \qquad \text{(D.2)}$$

with $x_k = k\Delta x = k/f_s$.

Suppose, first, that the frequency of the original continuous function $g(x)$ is exactly

$$f = \tfrac{m}{n} f_s \qquad \text{(D.3)}$$

In this case $p = \frac{n}{m}\Delta x$, meaning that we have exactly $\frac{n}{m}$ samples in each period p of $g(x)$, and the sampling step Δx is $\frac{m}{n}$ of the period p. The sampled signal we obtain in this case is:

$$g(x_k) = \sum_{l=-\infty}^{\infty} c_l \, e^{i2\pi l[(m/n)f_s]k/f_s} = \sum_{l=-\infty}^{\infty} c_l \, e^{i2\pi lk(m/n)} \qquad \text{(D.4)}$$

This corresponds, indeed, to the singular state of the (m/n)-order sub-Nyquist artifact, which is systematically plotted in Figs. 2 and on in row (a).

Now, suppose that the frequency of the given function $g(x)$ is not exactly $f = \frac{m}{n}f_s$, but rather:

$$f = \tfrac{m}{n} f_s + \varepsilon \qquad \text{(D.5)}$$

(where ε may be positive or negative). The sampling frequency f_s remains unchanged, so that we still have $x_k = k\Delta x = k/f_s$. The sampled signal is, in this case:

$$\begin{aligned} g(x_k) &= \sum_{l=-\infty}^{\infty} c_l \, e^{i2\pi l[(m/n)f_s + \varepsilon]k/f_s} \\ &= \sum_{l=-\infty}^{\infty} c_l \, e^{i2\pi l[k(m/n) + \varepsilon k/f_s]} \\ &= \sum_{l=-\infty}^{\infty} c_l \, e^{i2\pi l[x_k\varepsilon + \phi]} \qquad \text{with } \phi = k\tfrac{m}{n} \\ &= \sum_{l=-\infty}^{\infty} c_l \, e^{i2\pi l\varepsilon[x_k + (\phi/\varepsilon)]} \end{aligned} \qquad \text{(D.6)}$$

As we can see by comparing with Eqs. (D.1) and (D.2), this is a sampled version of the counterpart of $g(x)$ having frequency ε (i.e. period $1/\varepsilon$) and a nominal shift of $\frac{\phi}{\varepsilon}$, i.e. a *relative shift* of ϕ periods (Appendix B gives the definition of this term, and shows that in terms of ϕ Eq. (D.6) is cyclic modulo 1).

This means that:

(1) For integer $\phi = k\frac{m}{n}$, namely for $k = 0, n, 2n, 3n, \ldots$ we have:

$$g(x_k) = \sum_{l=-\infty}^{\infty} c_l \, e^{i2\pi l\varepsilon[x_k + 0]}$$

This signal has a shift of 0 (or of an integer multiple of its period $\frac{1}{\varepsilon}$).

(2) For $k = 1, n{+}1, 2n{+}1, 3n{+}1, \ldots$ we have:

$$g(x_k) = \sum_{l=-\infty}^{\infty} c_l \, e^{i2\pi l\varepsilon[x_k + (m/n)(1/\varepsilon)]}$$

This signal has a shift of $\frac{m}{n}$ times its period $\frac{1}{\varepsilon}$.

(3) For $k = 2, n{+}2, 2n{+}2, 3n{+}2, \ldots$ we have:

$$g(x_k) = \sum_{l=-\infty}^{\infty} c_l \, e^{i2\pi l\varepsilon[x_k + 2(m/n)(1/\varepsilon)]}$$

This signal has a shift of $2\frac{m}{n}$ times its period $\frac{1}{\varepsilon}$.

. . .

(n) For $k = n{-}1, 2n{-}1, 3n{-}1, 4n{-}1, \ldots$ we have:

$$g(x_k) = \sum_{l=-\infty}^{\infty} c_l \, e^{i2\pi l\varepsilon[x_k + (n-1)(m/n)(1/\varepsilon)]}$$

This signal has a shift of $(n-1)\frac{m}{n}$ times its period $\frac{1}{\varepsilon}$.

This means that the successive sampled points of our original function, $g(x_k)$, $k = 0, 1, 2, \ldots$ fall intermittently on one of n interlaced $g(x)$-shaped curves (that we call *envelopes*), which have all the same frequency ε and period $1/\varepsilon$, and which only differ from each other in their phase. More precisely, these n envelopes only differ from each other by successive relative shifts of $\frac{m}{n}$ periods $1/\varepsilon$, i.e. by successive shifts of $a = \frac{m}{n\varepsilon}$. This corresponds, indeed, to the situation we see in our figures in rows (b)-(c).

We have thus obtained Theorem 6.1.

References (for the main paper and the appendices)

[1] D. P. Mitchell and A. N. Netravali, "Reconstruction filters in computer graphics," Proceedings of SIGGRAPH 1988, *Computer Graphics*, 22 (1988) 221–228.

[2] J. D. Foley, A. van Dam, S. K. Feiner and J. F. Hughes, *Computer Graphics: Principles and Practice.* Addison-Wesely, Reading, Massachusetts, 1990 (second edition).

[3] G. Fielding, R. Hsu, P. Jones and C. DuMont, "Aliasing and reconstruction distortion in digital intermediates," *SMPTE Motion Imaging Journal*, 115 (2006) 128–136.

[4] G. L. Williams, "Sub-Nyquist distortions in sampled data, waveform recording, and video imaging," NASA Technical Memorandum TM-2000-210381, Ohio, 2000.

[5] M. Schwartz, *Information, Transmission, Modulation and Noise*. McGraw-Hill, NY, 1990 (fourth edition).

[6] I. Amidror, *The Theory of the Moiré Phenomenon, Volume I: Periodic Layers*. Springer, NY, 2009 (second edition).

[7] I. Amidror, *Mastering the Discrete Fourier Transform in One, Two or Several Dimensions: Pitfalls and Artifacts*. Springer, NY, 2013.

[8] I. Amidror and R. D. Hersch, "The role of Fourier theory and of modulation in the prediction of visible moiré effects," *Journal of Modern Optics*, 56 (2009) 1103–1118.

[9] E. O. Brigham, *The Fast Fourier Transform and Its Applications.* Prentice-Hall, NJ, 1988.

[10] J. D. Gaskill, *Linear Systems, Fourier Transforms, and Optics*. John Wiley & Sons, NY, 1978.

[11] R. W. Ramirez, *The FFT: Fundamentals and Concepts*. Prentice Hall, NJ, 1985.

[12] E. W. Weisstein, *CRC Concise Encyclopedia of Mathematics*. CRC, Boca Raton, 1999.

[13] D. L. Donoho, "Compressed sensing," *IEEE Transactions on Information Theory*, 52 (2006) 1289–1306.

[14] E. J. Candès, "Compressive sampling," *Proceedings of the International Congress of Mathematicians,* Madrid (2006) 1433–1452. http://statweb.stanford.edu/~candes/papers/CompressiveSampling.pdf (accessed August 22, 2014).

[15] R. Eschbach, "Generation of moiré by nonlinear transfer characteristics," *Jour. of the Optical Society of America A,* 5 (1988) 1828–1835.

[16] I. Amidror, "Tutorial: Sub-Nyquist artifacts and sampling moiré effects," Technical Report No. 203756, EPFL, Lausanne, 2015. Available online at: http://infoscience.epfl.ch/record/203756 (accessed February 14, 2015).

[17] R. N. Bracewell, *The Fourier Transform and its Applications*. McGraw-Hill, Boston, 2000 (third edition).

[18] D. M. Kammler, *A First Course in Fourier Analysis*. Cambridge University Press, Cambridge, 2007 (revised edition).
