# Supplementary material for: Sub-Nyquist artefacts and sampling moiré effects
Source: R Soc Open Sci. 2015 Mar 18;2(3):140550. doi: 10.1098/rsos.140550 (PMC4448827; doi:10.1098/rsos.140550)
Supplement: User s guide for the interactive applications. [file rsos140550supp6.pdf]

User's guide for the interactive Mathematica® applications `f_sweep_dense` and `f_sweep_env`

Two interactive Mathematica® applications accompany the article "Sub-Nyquist artifacts and sampling moiré effects" by Isaac Amidror: **`f_sweep_dense`** and **`f_sweep_env`**. These applications allow one to dynamically observe artifacts that may occur when sampling a continuous periodic signal $g(x)$ (cosine wave, square wave or sawtooth wave), while letting the signal's frequency f sweep along the frequency axis. The *sampling frequency* remains constantly fixed at $f_s = 8$. In each case, both a signal-domain panel and a Fourier spectral-domain panel are displayed simultaneously. The two applications are equivalent, but the second one is more sophisticated: In the signal-domain panel it shows both the original continuous signal and its sampled version, and in the spectral-domain panel it shows both the CFT (continuous Fourier transform) of the original continuous signal and the DFT (discrete Fourier transform) of the sampled signal.

1. Introduction

Sampling artifacts are best appreciated and understood by means of hands-on experimentation. The figures in the main article only show a limited number of particular cases that were carefully chosen to best illustrate the text. But a truly interactive manipulation of the various parameters can provide a much deeper insight into the dynamics of the phenomena in question. In particular, letting the frequency f of the original continuous signal sweep along the frequency axis, while keeping the sampling frequency f_s fixed, can offer a panoramic view of the various phenomena in question and clearly illustrate their dynamic evolution.

For this reason, a dedicated interactive software has been developed to accompany the main article. By manipulating the different parameters one may obtain a vivid graphic demonstration of the various sampling artifacts in question and of their dynamic behaviour, both in the signal domain and in the Fourier spectral domain.

Two versions of the software are provided:

1. **`f_sweep_dense.m`** – A simple interactive application that shows the sampling results as they would be observed on an oscilloscope, as well as their DFT (see Figs. C1-C2 in Appendix C). Historically, sub-Nyquist artifacts were first observed on an oscilloscope, and this version of the application will allow you "rediscover" these phenomena in a similar manner: Let the frequency f of the continuous periodic signal slowly sweep along the frequency axis, and observe the resulting sampled signal as if it were displayed on an oscilloscope.

2. **`f_sweep_env.m`** – A more sophisticated interactive application that allows one to see in more detail both the original continuous signal (plotted in continuous green lines) and its sampled version (discrete blue dots), as well as the modulating envelopes belonging to the corresponding sub-Nyquist artifacts. The spectral domain panel shows simultaneously the CFT of the original continuous signal (plotted in continuous cyan and magenta lines for the real and imaginary parts, respectively) and the DFT of the sampled signal (discrete cyan and magenta dots for the real and imaginary parts, respectively).

These two applications are written in the Mathematica® programming language. The Mathematica® sourse code is also provided, to allow users make their own experiments by modifying other parameters inside the source programs (such as the sampling frequency, the original periodic waves to be sampled, etc.). Users who do not possess the Mathematica® software will be able to run the enclosed CDF version of these two applications (**`f_sweep_dense.cdf`** and **`f_sweep_env.cdf`**) using the free CDF player that can be downloaded from the Wolfram CDF web page; but in this case no modifications can be done in the source code itself. To run an "**`.m`**" file click on the filename and then on the "Run Package" button at the top of the frame; to run a "**`.cdf`**" file click on the filename and then, if required, on the "Enable Dynamics" button at the top. If the source code is displayed, scroll down to see the graphics.

In both of these applications the same conventions are followed as in the main article. In particular, as explained in Sec. 4 of the article, the sampling frequency always remains fixed at $f_S = 8$ (thus giving a fixed sampling step of $\Delta x = 1/f_S = 0.125$). The number of sampling points is $N = 64$ in **`f_sweep_env`** and 8 times higher in **`f_sweep_dense`** (meaning that the sampling range is also multiplied by 8). Since the physical plot dimensions remain identical in both applications, the sampled signal in the latter as well as its DFT are displayed 8 times more densely than in the former.

Note that all the cases shown in the static figures of the main article can be also visualized interactively by entering the respective parameters into one of these applications (**`f_sweep_dense`** for Figs. C1-C3 in Appendix C, and **`f_sweep_env`** for all the other figures in the main article). You can use these applications at any point while reading the main article or its appendices, in order to better visualize the cases under discussion, and for experimenting with any other cases you may wish to test.

2. How to use the applications

The buttons "***m/n***" and "$\boldsymbol{\varepsilon}$" allow you to easily reach some of the most typical artifacts, such as the (1/2)-sub-Nyquist artifact, the (1/3)-sub-Nyquist artifact, etc., with some preset values of ε. You can then watch their dynamic behaviour while fine-tuning the frequency slider "***f***", as explained in more detail below.

This direct-access method using the buttons "***m/n***" and "$\boldsymbol{\varepsilon}$" is only a convenient shortcut for reaching some preselected signal frequencies $f = (m/n)f_S + \varepsilon$, but the same results can be also obtained by moving the frequency slider to the appropriate positions.

A more systematic way to study the phenomena in question, in either of the two applications, consists of watching the sampling results while the frequency f of the original continuous periodic signal $g(x)$ is slowly being varied. The user can vary the frequency f of the original continuous signal between $f = 0$ and $f = 2.5f_S = 20$, by slowly moving the slider "$\boldsymbol{f}$" using the mouse.

A second slider named "$\boldsymbol{\phi}$" allows to slowly modify the relative phase ϕ of the original periodic signal between the values 0 and 1 (in terms of periods).

You can also use the button "**`signal`**" to choose a continuous signal to be sampled among three predefined periodic waves: A cosine wave (used by default), a square wave, or a sawtooth wave. The sawtooth signal, due to its asymmetric period, can be used to illustrate the influence of the sign of ε (i.e. whether the signal frequency f is located above or below the singular frequency of the (m/n)-sub-Nyquist artifact in question, $(m/n)f_S$). See Remark 3 in Appendix C.

An additional button named "**`env`**" is provided in the application **`f_sweep_env`**. This button allows to show the curves of the n modulating envelopes of the preselected (m/n)-sub-Nyquist artifact, whenever a "***m/n***" or "$\boldsymbol{\varepsilon}$" button is set. Note, however, that when the frequency f is updated manually, we may get farther away from the selected (m/n)-sub-Nyquist artifact, and the continuously updated envelopes belonging to the (m/n)-sub-Nyquist artifact in question may no longer be relevant (see Remarks 4 and 5 in Appendix C). New envelopes will be automatically plotted whenever a new preselected case is chosen, i.e. whenever a "***m/n***" or "$\boldsymbol{\varepsilon}$" button is set.[1]

Remark: In order to move a slider more slowly (which may be very useful for fine tuning), simply hold down the Alt key (Windows) or the Option key (Macintosh) while dragging the mouse left or right. If you move the mouse outside the area of the slider, the value will move slowly in that direction as long as the mouse remains clicked. If you wish to move the slider even more slowly, you can hold down the Shift or Ctrl keys, or both, in addition to the Alt/Option key. This is particularly useful for dynamically observing the behaviour of sub-Nyquist artifacts that are only visible in a very small range of frequencies around their singular frequency. It is also possible to enter numerically any desired value (within the permitted range): Simply enter the desired numerical value into the small box to the right of the slider; when hitting the Enter key the application will update the display accordingly. For example, when setting the

[1] Note that "**`env`**" = **`yes`** is only meaningful in conjunction with a preselected (m/n)-sub-Nyquist artifact, i.e. after "***m/n***" and "$\boldsymbol{\varepsilon}$" have been assigned values. The application shows the envelopes that correspond to these selected values, and it continues updating them correctly even when f has been modified manually using the slider "$\boldsymbol{f}$", and even if *de facto* another (m/n)-sub-Nyquist artifact is currently being displayed. For example, if you set the buttons "***m/n***" = 1/3 and "$\boldsymbol{\varepsilon}$" = 1/8, and then manually move the "$\boldsymbol{f}$" slider to $f = 3.9$, you will still see the envelopes of the (1/3)-sub-Nyquist artifact, as they look at $f = 3.9$, although the dominant effect visible in the sampled signal at $f = 3.9$ is the (1/2)-sub-Nyquist artifact. In such situations the plotted envelope curves of the old (m/n) value no longer correspond to the envelopes of the currently displayed sub-Nyquist artifact. This application *does not* automatically identify the new (m/n)-sub-Nyquist artifact being displayed, and it always shows the envelopes of the last selected (m/n)-case, even if they are no longer relevant. This is done intentionally, in order to let you follow the evolution of the envelopes of the specified (m/n)-sub-Nyquist artifact even when f gets farther away from the corresponding singular point $(m/n)f_S$.

relative phase to $\phi = 0.75$ the original periodic function will be shifted to the left by three quarters of its period; in the particular case of the cosine signal, this simply gives a sine signal.

Remark: If you wish to increase or decrease the size of the displayed area, click with the mouse anywhere inside the figure; this will display a new thin gray frame around the entire displayed area, whose borders can be dragged with the mouse to obtain the desired dimensions.

3. Examples

The default parameter values are set to show a typical case, the (1/3)-sub-Nyquist artifact. Thus, when you enter the application **`f_sweep_dense`** you will get the following display, showing the sampled version of the cosine signal $g(x) = \cos(2\pi fx)$ with $f = (1/3)f_s + \varepsilon$, as well as its DFT:

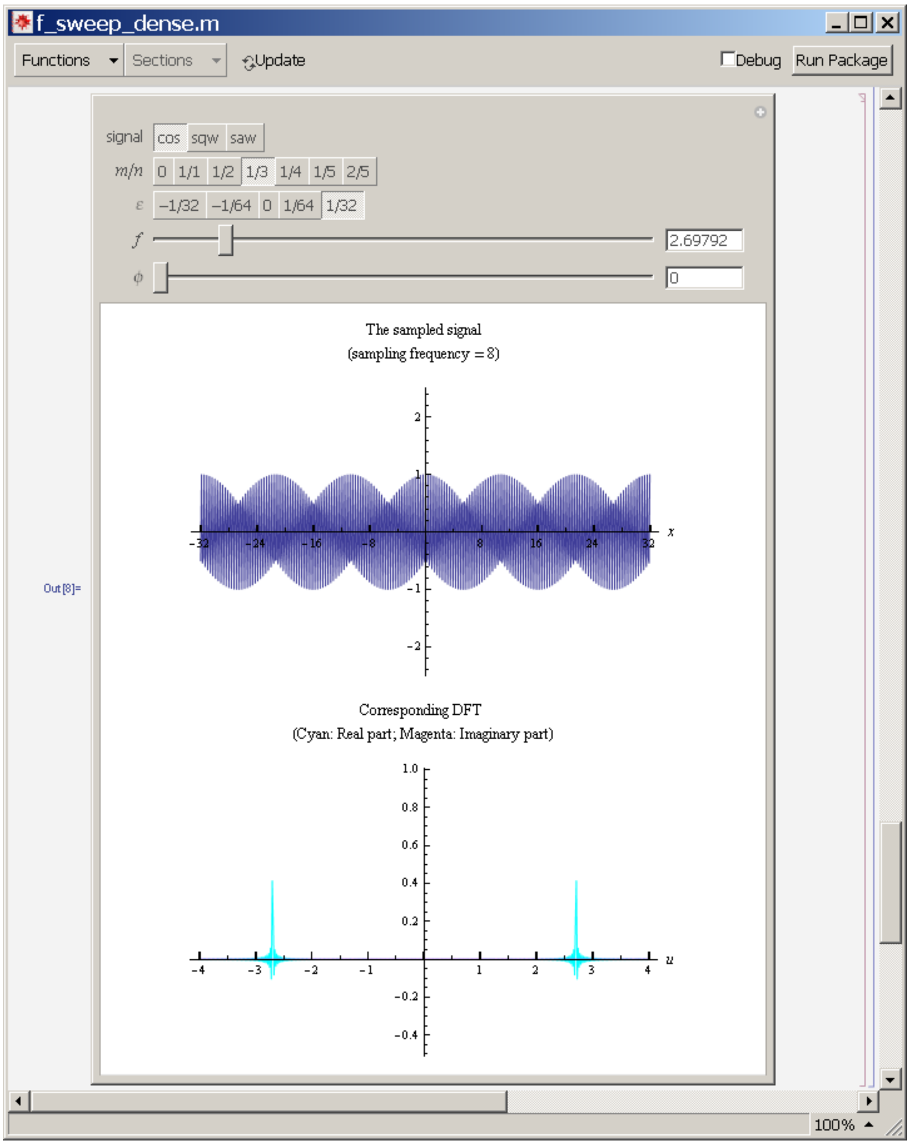

And when you enter the application **`f_sweep_env`** you will get the following display, showing the original and the sampled cosine signal as well as their respective CFT and DFT:

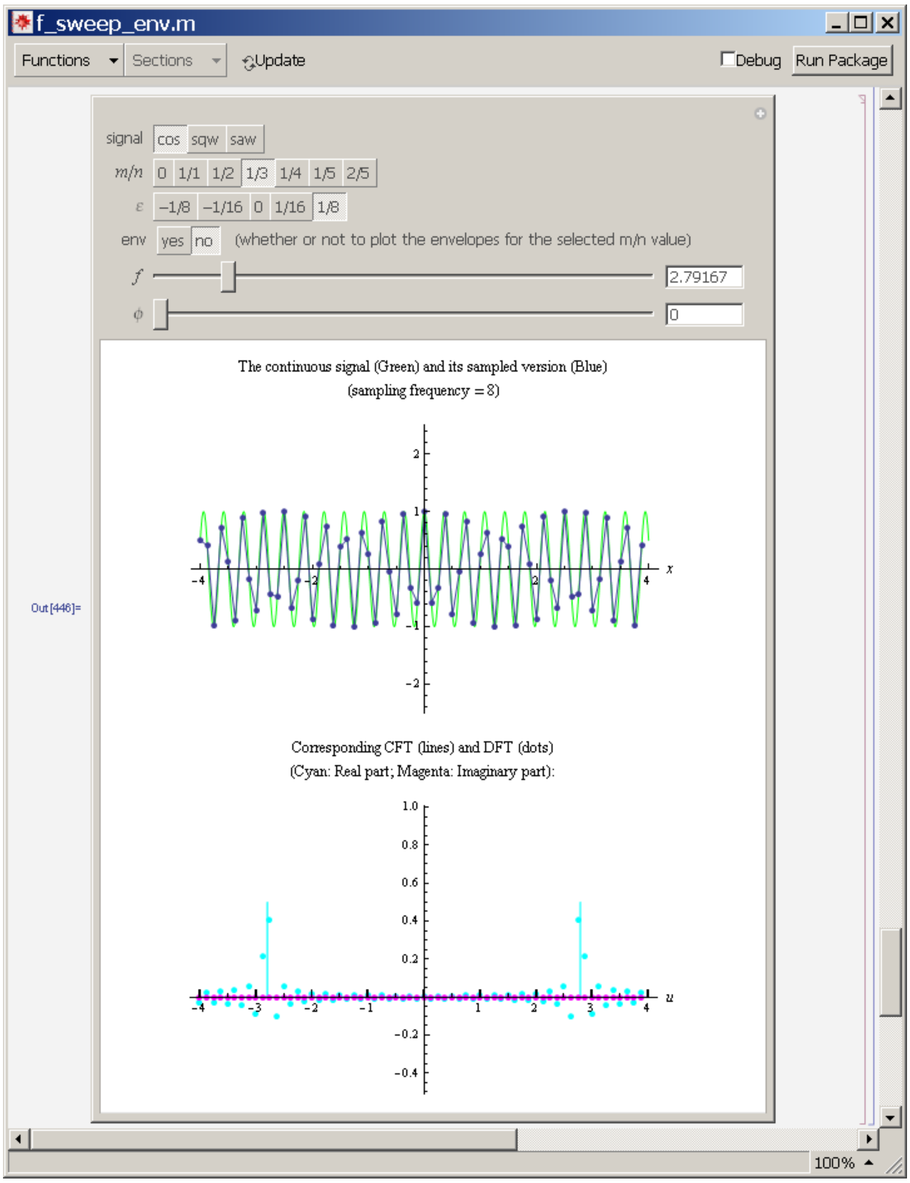

You may now try to view the same sub-Nyquist artifact with another preset value of ε, or select another (m/n)-sub-Nyquist artifact by clicking on a different m/n value. This is even more interesting when the "**`env`**" button is set to "yes" and the envelope curves are being displayed (in the application **`f_sweep_env`** only). When you select a new m/n or ε value, the frequency will immediately jump to $f = (m/n)f_S + \varepsilon$, and the new sub-Nyquist artifact will be displayed. Alternatively, you can simply sweep along the frequency axis by slowly moving the "f" slider, and observe the sampled signal and its

various artifacts as they appear and disappear each time f approaches and passes across a new singular point $(m/n)f_s$.

It is fun to watch how one shape gradually turns into another while slowly sweeping along the frequency axis (i.e. when gradually moving the frequency slider). In order to identify an unknown sub-Nyquist artifact that you encounter on your way (i.e. in order to determine its m,n values), follow the instructions given in Remark 6 (see Appendix C).

4. A guided tour along the frequency axis

The subject presented in the main article may seem at first view rather perplexing and counter-intuitive. It may be therefore didactically helpful to develop an intuitive understanding of the phenomena in question before delving into the formal explanations.

For this end, we need a much more detailed version of Fig. 1 of the main article. Fig. 1 provides a limited number of "snapshots" showing what happens to the sampled cosine signal and to its spectrum as the cosine frequency f is gradually varied between $f = 0.25$ and $f = 7.75$, while the sampling frequency remains fixed at $f_s = 8$. This sweep along the frequency axis can be done in a much more detailed and effective way using any of the two interactive applications provided here. You can slowly vary the cosine frequency f starting from $f = 0$ upward, and observe what happens to the sampled signal and to its spectrum each time you approach one of the frequencies f shown in Fig. 1.

As we can see during this stroll along the frequency axis, as long as f is small, the sampled cosine nicely follows the original continuous cosine, and no surprises occur in the spectra, too. At the other extreme case of Fig. 1, when f gets very close to $f_s = 8$, there are no surprises, either: Since f largely exceeds the Nyquist frequency $0.5f_s = 4$, aliasing (folding-over) occurs, and if the folded-over frequencies are very low (close to the spectrum origin), a low-frequency alias (sampling moiré) is clearly seen in the sampled signal, in the form of a low-frequency cosine. This folded-over low frequency is also clearly visible in the DFT of the sampled signal.

Now, let us concentrate on the situation when f is slightly below $0.5f_s$. As we can clearly see, a new low-frequency beating (or modulation) effect appears in the sampled signal, which is very different from the low-frequency moiré effect we see when f is very close to f_s. Consider, for example, the case of $f = 3.75$ (row (b) in Fig. 1): Here, we clearly see in the sampled signal a low-frequency effect with a period of 2 (in fact, the period of the modulating envelope of this beating effect is 4). However, looking at the spectrum, we see that no corresponding low frequency (neither 1/2 nor 1/4) is present. The only frequency which appears in the DFT is the relatively high frequency $f = 3.75$ of the original cosine, that is located close to the high-end frequency ($f = 4$) of the DFT. Furthermore, in the signal domain, the beating effect we see does not really correspond to a low-frequency signal, but rather to a highly oscillating signal that is only *modulated* by a low-frequency envelope. This is clearly not an aliasing (or moiré) effect, since no corresponding low frequency exists here. Note that as f approaches the critical (singular)

point $0.5f_S$ the period of the beats becomes bigger and bigger, until the beats completely disappear at exactly $0.5f_S$. But then, as f exceeds beyond $0.5f_S$, the beats reappear once again, and as f gets farther away from $0.5f_S$ their period becomes smaller, until they finally fade out and disappear. As explained in the main article, this phenomenon is the (1/2)-order sub Nyquist artifact, which occurs when f is close to the singular point $(1/2)f_S$, i.e. when $f = (1/2)f_S + \varepsilon$ (for any small positive or negative ε).

Thus, using the present applications, a *much more detailed* version of Fig. 1 can be obtained, illustrating a stroll along the frequency axis with much smaller steps Δf between successive "snapshots". It is highly instructive to observe, both in the signal and spectral domains, what happens as you sweep along the frequency axis. Note that during this sweep you will encounter all of the cases shown in Figures 2-4 of the main article, each time f approaches the corresponding critical frequency: $(1/3)f_S = 2.666...$ for Fig. 4, $(1/2)f_S = 4$ for Fig. 3, $(1/1)f_S = 8$ for Fig. 2, etc.

When you sweep through the range between $f = 0$ and $f = 0.5f_S$ more slowly, using smaller steps, you may discover new sub-Nyquist artifacts that you could have missed while sweeping with a coarser step. As explained in the main article, all of these beating phenomena occur around critical (singular) frequencies having the form $(m/n)f_S$. For example, Fig. 3 of the main article shows the (1/2)-order sub-Nyquist artifact which occurs around the frequency $f = (1/2)f_S = 4$, and Fig. 4 shows the (1/3)-order sub-Nyquist artifact which occurs around the frequency $f = (1/3)f_S = 2.666...$ Other similar beating artifacts occur for any integer ratio (m/n), but for bigger values of m,n they may be hardly visible (or simply obscured by stronger nearby (m/n)-order artifacts belonging to lower m,n values).

It is important to note that depending on the values of m,n such (m/n)-order artifacts may also occur *above* the Nyquist frequency of $0.5f_S$, where aliasing (folding-over) *does* exist. This is explained in Remark 8 (see Appendix C).

Remark: The CFT and DFT definitions being used in these applications follow the same conventions as in the main paper (see Chapter 2 in Ref. [7]):

$$G(u) = \int_{-\infty}^{\infty} g(x)\, e^{-i2\pi ux}\, dx \qquad -\infty < u < \infty$$

$$G(n) = \sum_{k=0}^{N-1} g(k)\, e^{-i2\pi kn/N} \qquad n = 0, \dots, N-1$$
