# Supplementary material for: Sub-Nyquist artefacts and sampling moiré effects
Source: R Soc Open Sci. 2015 Mar 18;2(3):140550. doi: 10.1098/rsos.140550 (PMC4448827; doi:10.1098/rsos.140550)
Supplement: Glossary of the main terms. [file rsos140550supp7.pdf]

Glossary of the main terms

This glossary accompanies the article "Sub-Nyquist artifacts and sampling moiré effects" by Isaac Amidror. It explains the main terms being used in the article, and demonstrates them visually using the interactive applications **`f_sweep_dense`** and **`f_sweep_env`**. Throughout this glossary $g(x)$ represents a continuous periodic signal having frequency f, $g(x_k)$ represents the sampled signal, and f_s is the sampling frequency. The suggested interactive demonstrations are presented in blue type.

aliasing (or *folding over*) –

When an original continuous signal (periodic or not) is sampled at a frequency f_s which does not satisfy the Nyquist condition of the sampling theorem, i.e. when f_s is not at least twice the highest frequency contained in the original continuous signal, the signal's frequencies above $0.5f_s$ (and below $-0.5f_s$) fold-over into the frequency range $-0.5f_s \ldots 0.5f_s$. In other words, the resulting sampled signal contains new false frequencies within this range, which do not exist in the original signal. This phenomenon is known as *aliasing*.

As a simple interactive demonstration, let us visualize dynamically what happens when sampling the continuous signal $g(x) = \cos(2\pi fx)$, as the signal's frequency f exceeds the Nyquist frequency $0.5f_s$ (so that $f_s < 2f$). For this end, enter the application **`f_sweep_env`** and select there "**`signal`**" = **`cos`** and "**`env`**" = **`no`**. Then, manually set the frequency f to the value 3, which is still below the Nyquist frequency $0.5f_s = 4$ (remember that in all our figures and applications we always use $f_s = 8$). Now, let the frequency f slowly increase, and watch what happens in the spectral domain. (To slow down the movement of the "***f***" slider, hold down the Alt or Option key while dragging the slider with the mouse; see the user's guide of the applications). As long as the frequency f is located below the Nyquist frequency $0.5f_s = 4$, the DFT of the sampled signal follows the impulses of the original CFT (possibly with some leakage, which only occurs when f is located between two successive discrete frequencies of the DFT; see, for example, Chapter 6 in [7]). But when the frequency f goes beyond the Nyquist frequency $0.5f_s = 4$, the corresponding frequencies in the DFT are folded over, and they re-enter into the range $-0.5f_s ... 0.5f_s$ of the DFT spectrum in the opposite direction. These false lower frequencies in the DFT (which do not exist in the original continuous signal $g(x)$ and in its CFT) are the spectral-domain manifestation of aliasing. In the signal domain, aliasing manifests itself by "mimicking" the correct cosine signal with a false, lower-frequency cosine signal that passes through the same sampling points. For more details on aliasing and how it affects the signal and spectral domains see, for example, Chapter 5 in [7].

sampling moiré effect –

An artifact which may occur due to aliasing when sampling a periodic signal $g(x)$. It consists of a new false low-frequency f_M which appears in the sampled signal $g(x_k)$ and in its spectrum, although it does not exist in the original signal $g(x)$. When the new frequency f_M is very low (very close to the spectrum origin) the moiré effect is highly visible, but as f_M increases the moiré gradually becomes less conspicuous, until it finally completely disappears. Note that in order for the moiré effect to be clearly visible, f_M must be much smaller than the signal frequency f and the sampling frequency f_s.

A sampling moiré effect occurs in the sampled signal whenever f approaches an integer multiple of f_s, i.e whenever $mf_s - f \approx 0$, $m = 1,2,\ldots$ In each of these cases a new low frequency $f_M = mf_s - f$ is generated in the spectrum of the sampled signal, close to the spectrum origin. However, if $g(x)$ is a periodic function having in its spectrum two harmonics of its frequency f, a sampling moiré effect may also occur whenever $mf_s - 2f \approx 0$, $m = 1,2,\ldots$ since in these cases too a new false low frequency, $f_M = mf_s - 2f$, is generated close to the spectrum origin. The extension to functions $g(x)$ having higher harmonics of f is straightforward.

In general, the sampling moiré effect which occurs when $mf_s - nf \approx 0$ is called a $(m,\text{-}n)$-moiré. It corresponds to the new low frequency $f_M = mf_s - nf$, which is generated in the sampled signal due to the interaction between the m-th harmonic of the sampling frequency f_s and the n-th harmonic of f (provided that $g(x)$ has a non-zero n-th harmonic).

As a simple interactive demonstration, let us visualize a few sampling moiré effects that may occur when sampling the continuous signal $g(x) = \cos(2\pi fx)$. For this end, enter the application **`f_sweep_env`** and select there "**`signal`**" = **`cos`**, "***m*/*n***" = 1/1 and "***ε***" = 1/8. This shortcut brings you directly to the sampled cosine at the frequency $f = (1/1)f_s + 1/8$, where f is close to $f_s = 8$ (see the figure on next page). The sampled signal we see here presents a highly visible moiré effect with period 8 and frequency 1/8 (compare the original signal shown in green lines with its sampled version shown by the discrete dots). Note that the spectral domain, too, contains here a new very low frequency of 1/8, which does not exist in the CFT of the original signal $g(x)$.

It is highly instructive to slowly vary the frequency f to both directions, using the "***f***" slider, in order to observe how this affects our moiré effect.

A very similar moiré effect occurs also when the frequency f approaches the value $2f_s = 16$ (try this and see!), or any other integer multiple of f_s.

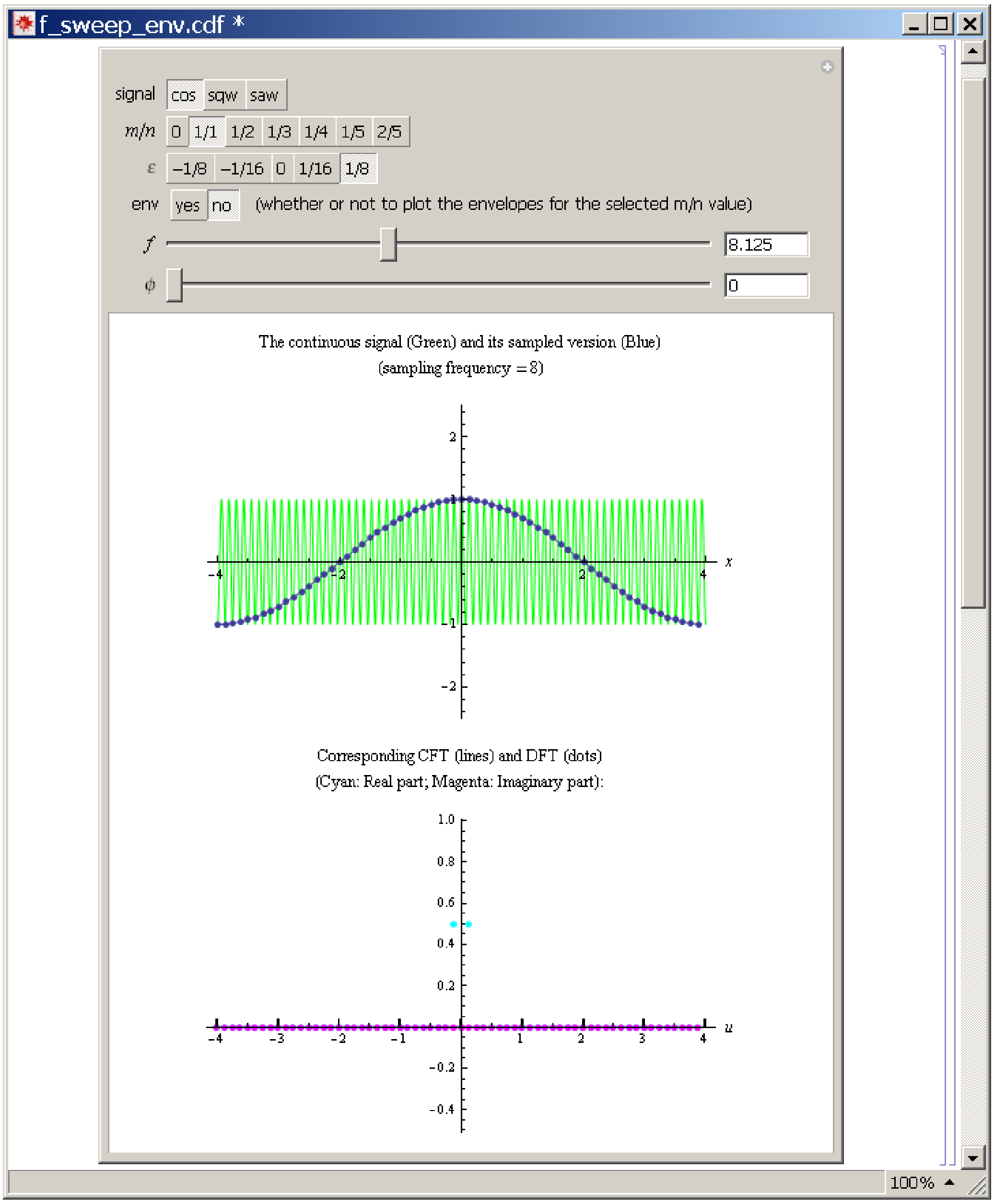

sub-Nyquist artifact –

A beating artifact which may occur when sampling a periodic signal $g(x)$ of frequency f whenever the frequencies f and f_s satisfy $f \approx (m/n)f_s$ with integer m,n ($n > 1$). The beating artifact which occurs for a given reduced ratio m/n is called a (m/n)-order sub-Nyquist artifact. Sub-Nyquist artifacts have several intriguing properties:

(a) They may appear where the Nyquist condition is fully satisfied, so that no aliasing or sampling moiré artifacts should be present.

(b) Unlike in aliasing or moiré phenomena, the periods (or frequencies) of these beating artifacts are not represented in the Fourier spectrum, although they are clearly visible in the sampled signal.

(c) Furthermore, in the signal domain, the beating effect in question does not really correspond to a low-frequency signal, but rather to a highly oscillating signal that is only *modulated* by low-frequency envelopes.

Sub-Nyquist artifacts with $m/n < 1/2$ are most interesting, since they occur below the Nyquist frequency. But other cases may also occur when $m/n > 1/2$, where aliasing does exist.

As an illustration, let us dynamically visualize a few sub-Nyquist artifacts that may occur when sampling the continuous signal $g(x) = \cos(2\pi fx)$. For this end, enter the application **f_sweep_dense** and select there "**signal**" = **cos**, "***m*/*n***" = 1/2 and "***ε***" = -1/32. This shortcut brings you directly to the sampled cosine at the frequency $f = (1/2)f_s - 1/32$, where f is slightly below $(1/2)f_s = 4$ (see the figure on next page). This sampled signal illustrates the highly visible (1/2)-order sub-Nyquist artifact. Note that this artifact is *not* a sampling moiré effect, but indeed a sub-Nyquist artifact: (a) it occurs where the Nyquist condition is fully satisfied and no aliasing or moiré effects may exist; (b) its low frequency is not represented in the spectral domain (note that the DFT only contains the relatively high frequency f of the original cosine, slightly below the highest possible frequency of the DFT, $0.5f_s = 4$); and (c) it consists of a highly oscillating signal, and not of a true low-frequency signal as in the previous figure. See the figure on next page, and compare it with the true moiré effect which occurs when you select "***m*/*n***" = 1/1 rather than "***m*/*n***" = 1/2 (compare the two cases with respect to the three properties (a)-(c)).

Here, too, it is highly instructive to slowly vary the frequency f to both directions, using the "***f*** " slider, in order to observe how this dynamically affects our sub-Nyquist artifact.

Finally, it is interesting to note that in functions $g(x)$ which possess a non-zero n-th harmonic nf of their frequency f, the (m/n)-order sub-Nyquist artifact turns into a sampling moiré effect: In such cases the low frequency $f_M = mf_s - nf$ does exist in the sampled signal $g(x_k)$ and in its spectrum; and yet, the highly oscillating nature of the sub-Nyquist artifact is still preserved. These "hybrid" cases are explained in Remark 9 (see Appendix C). To see such a hybrid case, select once again "***m*/*n***" = 1/2 and "***ε***" = -1/32, which brings you back to the (1/2)-order sub-Nyquist artifact, and then select "**signal**" = **sqw**. Note that unlike the cosine signal, the square wave possesses all the harmonics nf of its frequency f, including $2f$. And indeed, looking at the spectral domain, we see that this time the DFT of the sampled signal does contain low frequencies near the origin. But as expected, this does not occur in the cosinusoidal case, when you select "**signal**" = **cos**.

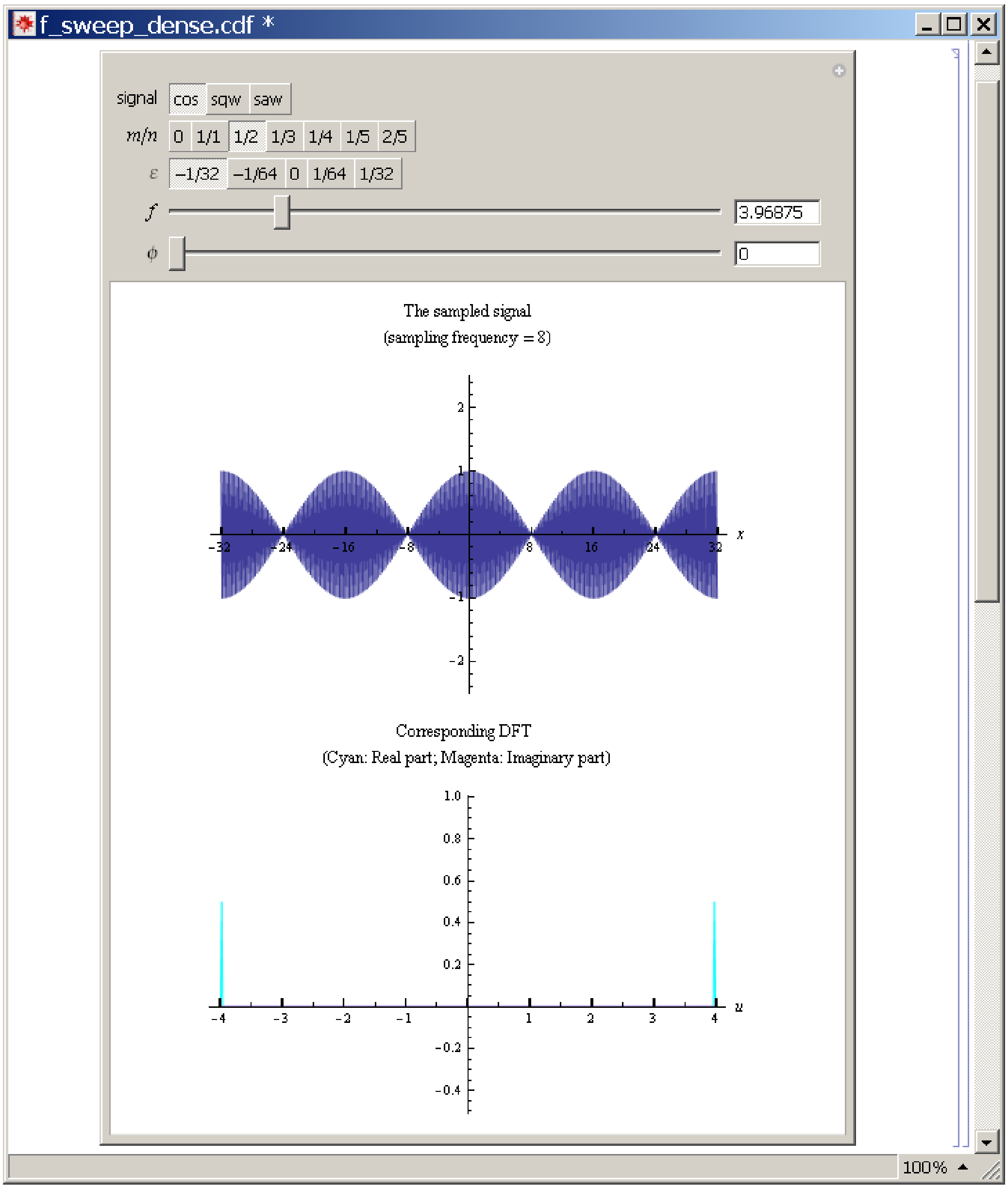

To see another example of a sub-Nyquist artifact, select "**signal**" = **cos**, "***m*/*n***" = 1/3 and "***ε***" = 1/32. This shortcut brings you directly to the sampled cosine at the frequency $f = (1/3)f_S + 1/32$, where f is very close to $(1/3)f_S = 2.666...$ (see the figure on next page). This sampled signal illustrates the highly visible (1/3)-order sub-Nyquist artifact. Note that this artifact is *not* a sampling moiré effect, but indeed a sub-Nyquist artifact: just as the previously discussed (1/2)-order sub-Nyquist artifact, the (1/3)-order sub-Nyquist artifact, too, fully satisfies the properties (a)-(c) mentioned above.

Once again, it is highly instructive to slowly vary the frequency f to both directions, using the "***f*** " slider, in order to observe how this affects our sub-Nyquist artifact.

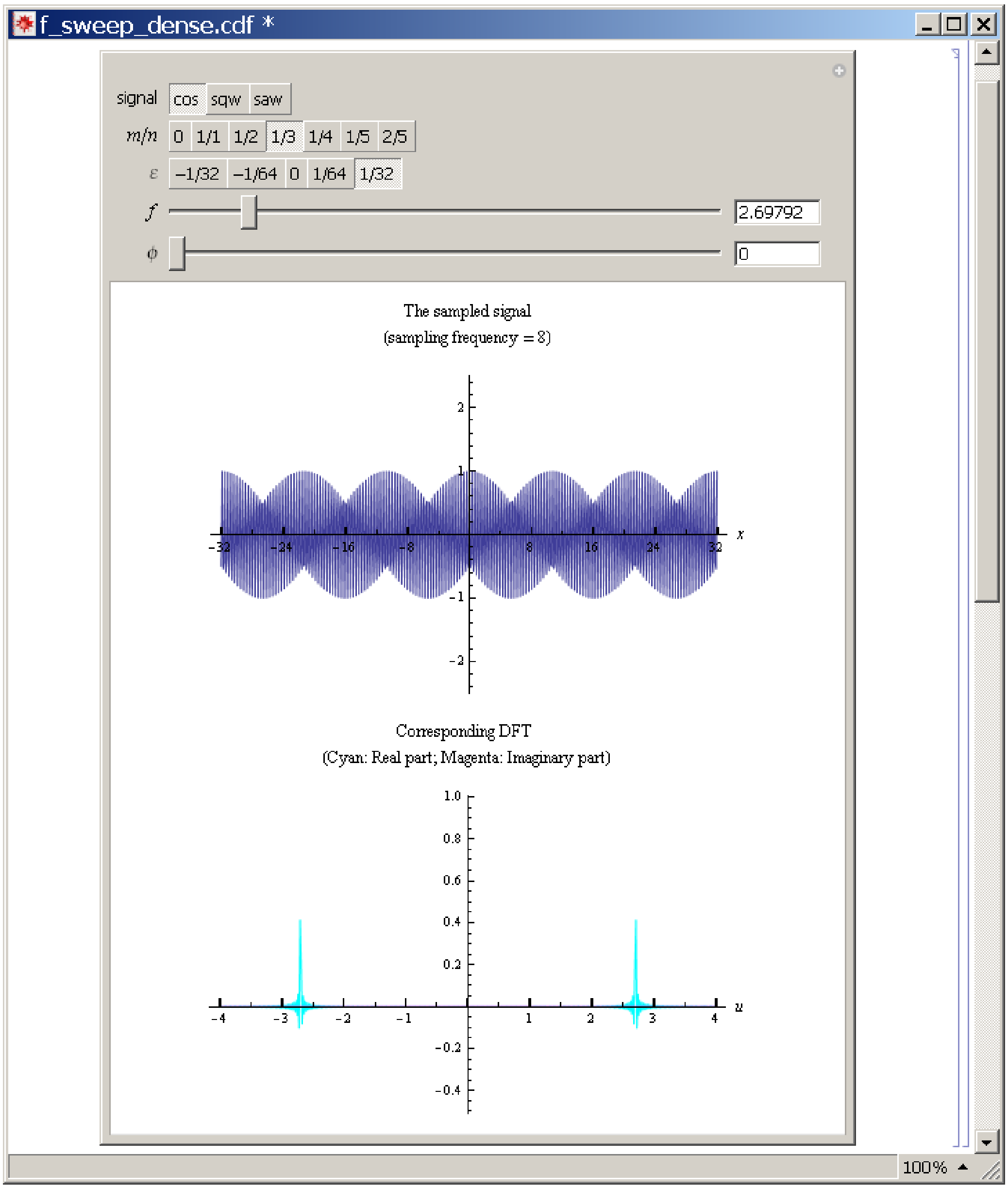

If you also want to see the original continuous signal and its CFT, in order to better understand what exactly happens when the original signal is being sampled, you may use the application **f_sweep_env** instead (see the figure below). This application also allows you to select "**env**" = **yes** in order to display the envelopes of the preselected sub-Nyquist artifact, and observe them as they dynamically evolve while you slowly vary the frequency *f*.

Note, however, that the sub-Nyquist artifacts look much more prominent when using the application **f_sweep_dense**. As explained in Remark 2 in Appendix C, the reason is twofold: On the one hand, the presence of the original continuous signal in the signal domain of the application **f_sweep_env** obscures the shape of the sampled signal. And on the other hand, the use in **f_sweep_dense** of a much denser display, in which the sampled points are connected by line segments (like on the display of an oscilloscope), further

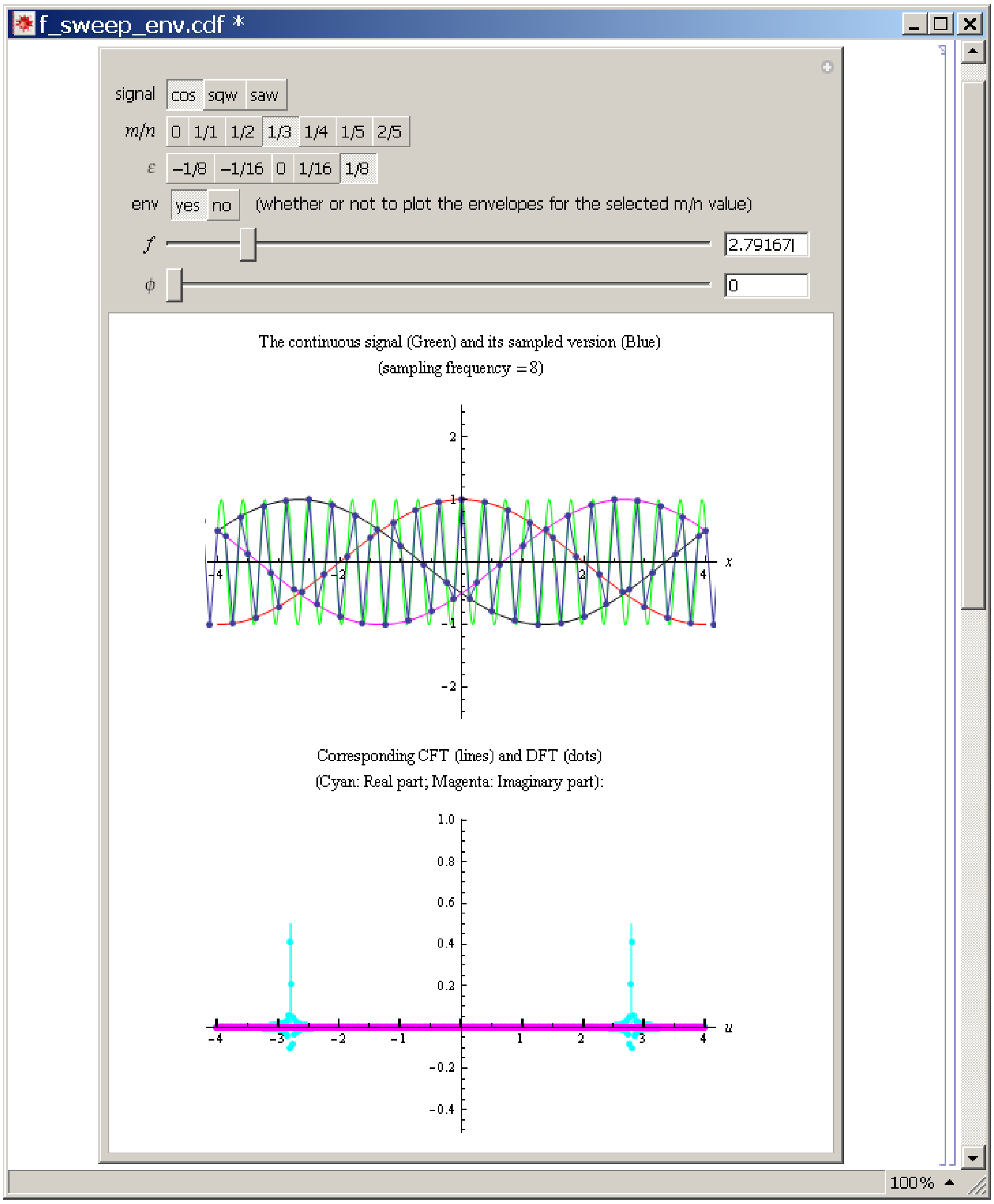

enhances the visibility of the artifact. For example, when selecting "***m/n***" = 1/5 or "***m/n***" = 2/5 in **`f_sweep_dense`** the resulting sub-Nyquist artifacts are clearly visible, while in **`f_sweep_env`** they are hardly recognizable without explicitly drawing their respective envelopes, by selecting "**`env`**" = **`yes`**.

singular state (or *singular point*) –

A singular state of a given sampling moiré effect (or sub-Nyquist artifact) is a situation in which the periodicity of the effect in question becomes infinitely large and momentarily disappears. This happens in a sampling moiré effect when f_M exactly equals 0, and in a (m/n)-order sub-Nyquist artifact when f exactly equals $(m/n)f_s$ (or in other words, when $\varepsilon = (m/n)f_s - f$ exactly equals 0). Each moiré effect or sub-Nyquist artifact has a unique distinct singular point.

singular frequency (or *critical frequency*) –

The signal frequency f at which a given moiré effect (or sub-Nyquist artifact) reaches its singular state. In both moiré effects and sub-Nyquist artifacts, as f approaches the singular frequency of the artifact in question, the artifact becomes larger and more conspicuous, until at the singular point itself the artifact becomes infinitely large and momentarily disappears. As f gradually pursues its way beyond the singular frequency, the artifact "comes back from infinity" and becomes again visible with a very large period. Then it starts getting smaller and smaller, until it finally fades out and disappears.

In order to visualize dynamically the behaviour of an artifact around its singular point, let us consider a few moiré or sub-Nyquist artifacts that may occur when sampling the continuous signal $g(x) = \cos(2\pi fx)$. For this end, enter the application **`f_sweep_dense`** and select there "**`signal`**" = **`cos`**, "***m*/*n***" = 1/1, and "***ε***" = 1/32. This shortcut brings you directly to the vicinity of the first-order moiré effect which occurs around $f = f_S$. Try to slowly vary the frequency f to both directions, and see how this affects the moiré effect (both in the signal and in the spectral domains). Then, select "***m*/*n***" = 1/3 and "***ε***" = 1/32. This shortcut brings you directly to the vicinity of the (1/3)-order sub-Nyquist artifact, which occurs around $f = (1/3)f_S$. Once again, try to slowly vary the frequency f to both directions, and see how this affects the sub-Nyquist artifact (in the signal domain as well as in the spectral domain). You can also try other pre-selected sub-Nyquist artifacts, such as the cases of (1/4), (1/5), or (2/5). If you wish to try any other (m/n)-order case, simply move f to the vicinity of $(m/n)f_S$, and observe there what happens in the signal and spectral domains while you slowly vary f.

sweeping (*along the frequency axis*) –

The action of letting the frequency f gradually vary along the frequency axis.

Such a stroll along the frequency axis is very instructive, and it can be easily done using any of the two provided interactive applications. Simply position the "***f***" slider at your desired starting point, and slowly drag it up or down along the frequency axis. For example, you may start your stroll at a very low frequency, such as $f = 0.25$, where the sampled signal almost perfectly follows the original continuous cosine, and watch what happens on your way as you slowly increase f. It is instructive to see how the various sub-Nyquist artifacts slowly appear and disappear, even when f is being varied well below the Nyquist frequency $0.5f_S = 4$. Note, for example, what happens when f approaches values such as $(1/4)f_S = 2$, $(1/3)f_S = 2.666...$ etc. In contrast, whenever f approaches an integer multiple of f_S, a true sampling moiré effect occurs in the sampled cosine. But in both cases, the dynamic behaviour of the artifact in question as f approaches the singular point and then passes beyond it is remarkably similar, as demonstrated above.
